# Optimising bifacial dye-sensitized solar cells with graphene-enhanced TPT configuration photoanode for operational stability

**DOI:** 10.1038/s41598-025-06097-4

**Published:** 2025-11-03

**Authors:** Hussein A. AlSultan, Suhaidi Shafie, Mohd Nizar Hamidon, Ismayadi Ismail, Shyam S. Pandey, Fauzan Ahmad

**Affiliations:** 1 Institute of Nanoscience and Nanotechnology (ION2), Universiti Putra Malaysia, Jalan UPM, Serdang, Selangor 43400 Malaysia; 2 Faculty of Engineering, Universiti Putra Malaysia, Jalan UPM, Serdang, Selangor 43400 Malaysia; 3 Department of Biological Functions Engineering, Kyushu Institute of Technology, Sensui-cho, Kitakyushu, Fukuoka 804-8550 Japan; 4 Malaysia-Japan International Institute of Technology, Universiti Teknologi Malaysia, Kuala Lumpur, Wilayah Persekutuan 54100 Malaysia

**Keywords:** Bifacial DSSC, Tri-layer structure, Photoanode, Graphene, Stability, Electrical and electronic engineering, Graphene, Nanoparticles, Solar energy and photovoltaic technology, Graphene

## Abstract

Bifacial Dye-sensitized solar cells (bifacial DSSCs) are a promising low-cost and with promising light absorbance alternative to conventional DSSCs, known for their potential for transparency and customizability to be integrated into building-integrated photovoltaic (BIPV). This study explores enhancing efficiency and stability in bifacial DSSCs by integrating graphene into the bifacial configured photoanode. Graphene was incorporated into titanium dioxide ($\hbox {TiO}_2$) photoanodes using a modified Ti-Nanoxide (T/sp) commercial paste, and powder type (P25) configurate as (T/sp-P25-T/sp) in tri-layer (identified optimal structure to maximizing bifacial light harvesting in our previous study) with Concentrations of 0.05%, 0.1%, and 0.2% graphene were tested. The structural, optical, and electrical properties of the materials were characterised using field emission scanning electron microscopy (FESEM), energy-dispersive X-ray spectroscopy (EDS), Raman spectroscopy, ultraviolet-visible (UV-Vis) spectroscopy, Tauc plots, current-voltage (J-V) measurements, and electrochemical impedance spectroscopy (EIS). The incorporation of 0.1% graphene resulted in the highest initial power conversion efficiency (PCE) of 11.09% under combined front and back illumination at the 4th day of testing, indicating improved electron transport and stability. Over a period of ten days, the performance of these cells remained significantly superior compared to those with pure $\hbox {TiO}_2$. These results highlight the potential of graphene-enhanced TPT-configured photoanodes for improving both the efficiency and durability of bifacial DSSCs, particularly in applications such as building-integrated photovoltaics.

## Introduction

Dye-sensitised solar cells have garnered significant attention due to their low production costs, straightforward fabrication, and abundant, non-toxic materials^1,2^. They are particularly noted for their efficiency under low-light conditions, potential for transparency, and colour customisation, making them a viable alternative to conventional silicon-based photovoltaics. DSSCs consist of a photosensitive dye, a semiconductor (typically Titanium Dioxide or $\hbox {TiO}_2$), an electrolyte, and counter electrodes. The operational mechanism involves dye molecules absorbing sunlight, exciting electrons from highest occupied molecular orbital (HOMO) to the lowest unoccupied molecular orbital (LUMO) energy state. These electrons are then transferred to the semiconductor’s conduction band, circulate through the external circuit, and return at the counter electrode, with electrolyte ions replenishing the dye’s electrons to maintain the cell’s operational cycle^3,4^.

Ruthenium-based complexes are fundamental to DSSCs due to their stability and suitable spectral properties, which, despite having lower molar extinction coefficients compared to typical donor-$\pi$-acceptor (D-$\pi$-A) organic dyes, still contribute to relatively high PCE and excellent anchoring to $\hbox {TiO}_{2}$ particles^5^. The design of these complexes, particularly the donor-$\pi$-acceptor, is critical for achieving high efficiencies, reaching up to 14.2% PCE^6^. Platinum (Pt) also plays a crucial role in enhancing DSSC performance through various methods such as doping, Pt-based counter electrodes (CEs), and Pt-decorated reduced graphene oxide (rGO) composites. These approaches have demonstrated improvements in efficiency due to reduced series resistance and enhanced electron transfer rates^7,8^.

Despite these advancements, DSSCs’ performance is often limited by the electron transport efficiency of $\hbox {TiO}_2$, which suffers from suboptimal electron-hole separation efficiency^9^. Tailoring the nanostructure and surface chemistry of $\hbox {TiO}_2$ for example by anodic nanotube growth followed by $\hbox {TiCl}_4$ post-treatment has been shown to lower series resistance and raise PCE by $\sim$27 %^10^. Similar mobility gains have been reported for cation-doped $\hbox {TiO}_2$ for instance, Zr-doped nanoparticles used as electron transport layers in perovskite cells cut charge-transfer resistance in half and boost $V_\text {OC}$ by 70mV^11^. Integrating graphene into $\hbox {TiO}_2$ matrix has shown promise in addressing these limitations by enhancing electron transport speed and reducing recombination rates, thereby improving photocatalytic activity and light absorption efficiency^12,13^.

Stability remains a critical challenge for DSSCs, as dye, electrolyte, and interfacial components degrade over time; recent studies show that encapsulating $\hbox {TiO}_2$ nanotubes with Cs nanoparticles, for example, can markedly suppress trap states and improve thermal durability^14^. Recent advancements have focused on developing stable materials and electrolyte formulations. For instance, water-containing $\hbox {I}^-$/$\hbox {I}_3^-$ redox electrolytes have maintained performance and stability even with high water content^15^. Gel polymer electrolytes (GPEs) based on hydroxypropyl cellulose (HPC) and MPII have shown high ionic conductivity and sustained efficiency over long periods^16^. Other studies have used Triton X-100 to improve dye adsorption on $\hbox {TiO}_2$ photoanodes, enhancing both current short circuit density ($\hbox {J}_{SC}$) and overall PCE^17^. Comprehensive reviews have identified key factors influencing stability and effective strategies for enhancing DSSC longevity^18^.

Bifacial DSSCs, capable of generating electricity from both sides, are notable for their performance under ambient light and aesthetic qualities like transparency and vibrant colours, making them suitable for building-integrated photovoltaics (BIPVs). Innovations in transparent counter electrodes using materials such as PEDOT and Ru-Se alloys have broadened their applicability^19–21^. Graphene, as a doping agent, has the potential to significantly enhance the PCE and durability of DSSCs due to its superior optoelectronic and mechanical properties^22,23^. Its versatility allows it to be used as a cathode support and an anode enhancer, improving electron transfer efficiency^24,25^. However, to our knowledge, integrating graphene in a trilayer structure photoanode in bifacial DSSCs has not yet been investigated. In our previous study^26^, a novel approach for bifacial DSSC of trilayer structure has been investigated. The configuration of the Ti-Nanoxide (T/sp) transparent layer for the first and third layers and the $\hbox {TiO}_2$ variant of (P25) as the light-scattering middle layer has resulted in the highest PCE for bifacial DSSCs due to higher light absorption from both sides. However, electron mobility has yet to be improved in the reported configuration. Modifications of graphene through doping, compositing, and coating with $\hbox {TiO}_2$ have led to significant performance improvements in DSSCs, which are marked by higher short-circuit current densities and enhanced PCEs. Integrating graphene in bifacial DSSCs could revolutionise back illumination and ambient light harvesting, marking a significant advancement in photovoltaic technology.

Building upon our prior research, this study aims to further enhance the efficiency and stability of DSSCs through strategic modifications to the Stack Formation Framework (SFF) trilayer structure and its configuration of (T/sp-P25-T/sp) or TPT^26^. The primary objectives are to improve electron mobility and enhance overtime working stability. We seek to optimise electron transport and reduce recombination losses by integrating graphene into the TPT configuration. This approach addresses key challenges in light harvesting and electron-hole separation, aiming to significantly improve the operational stability and efficiency of DSSCs under laboratory conditions. Moreover, this work distinguishes itself from previous studies by have doped $\hbox {TiO}_2$ with graphene to enhance single-side DSSC performance. to our knowledge this is the first demonstration of graphene?enhanced trilayer TPT configuration photoanodes in bifacial DSSCs, achieving over 11% combined PCE and dramatically improved 10-day stability under both front and back illumination.

## Materials and method of work

### Materials

The materials used in this study were selected based on their established efficacy in DSSC applications. The $\hbox {TiO}_2$ paste, Transparent Semiconductor Paste Ti-Nanoxide (Product Code: T/sp), and the Pt counter electrodes were sourced from Solaronix, known for their high quality in photovoltaic applications. $\hbox {TiO}_2$ nanoparticles were obtained as nanopowder (P25) from Degussa and processed into a paste in our lab due to their high surface area and photocatalytic properties. Graphene nanopowder (AO-3: 12nm flakes) nanosheets from graphene Supermarket were chosen for their superior electrical conductivity and large surface area. Transparent conducting oxide (TCO) glass substrates, specifically fluorine-doped tin oxide (FTO) glass with a sheet resistance of 15 $\Omega$/sq, provided a conductive base for the $\hbox {TiO}_2$ layers. Acetonitrile (solvent), iodine, lithium iodide, 4-tert-butylpyridine, and ethyl-methyl-imidazolium iodide were used for the electrolyte fabrication. All other chemicals, including solvents and reagents, were of analytical grade and used as received without further purification.

### Substrate preparation

The FTO glass substrates were prepared by cutting the glass into 2.5 cm x 2 cm dimensions and marking the non-conductive side to ensure proper orientation during subsequent processes. The cleaning process involved an initial rinse with tap water and detergent, followed by a 10-minute ultrasonication to remove any adhered particles. The substrates were then thoroughly rinsed with tap water to remove detergent residues. Following this, a systematic cleaning process using acetone, isopropyl alcohol (IPA), and ethanol was conducted, with each substrate immersed for 15 minutes with the conductive area facing upwards. Post-cleaning, the substrates were air-dried using a stream of hot air and subjected to UV-light treatment to remove any remaining organic residues and enhance the cleanliness of the conductive surface.

### $\hbox {TiO}_2$ paste preparation and graphene doping

In a modification from our last report on SFF^26^ in terms of adding graphene as a dopant material to enhance the electrochemical activity of the cell, the fabrication of the photoanode paste began with preparing an ethanol-ethyl cellulose mixture by dissolving 5 grams of ethyl cellulose in 10 mL of ethanol and stirring for two hours. Simultaneously, 1 gram of $\hbox {TiO}_2$ P25 was heated at 100 °C to remove residual moisture, adding 0.16 mL acetic acid. The mixture was then incrementally combined with 0.8 mL of deionised water and 10 mL of ethanol. Subsequently, another 10 mL of ethanol was pre-mixed with the desired percentage of graphene, and the mixture was ultrasonicated for one hour. Finally, 3.5 mL of $\alpha$-terpineol was added, and the combined solution was stirred for 12 hours and evaporated until the volume was reduced to one-tenth to achieve the desired paste viscosity for photoanode application.

### Electrolyte and counter electrode preparation

The electrolyte for the DSSCs was synthesised using the following components and concentrations: 5 mL of acetonitrile as the solvent, 0.05 M iodine ($\hbox {I}_2$) corresponding to 64 mg, 0.1 M lithium iodide (LiI) corresponding to 67 mg, 0.5 M 4-test-butyl pyridine (tBP) corresponding to 0.4 mL, and 0.6 M ethyl-methyl-imidazolium iodide (EMII) corresponding to 798 mg. These components were mixed in a 10 mL beaker and stirred using a magnetic stirrer for 15 minutes to ensure complete dissolution and homogeneity^27^. The resulting electrolyte solution was transferred into a well-sealed 10 mL bottle to prevent rapid evaporation. The counter electrodes were prepared using a ready-to-use Pt solution sourced from Solaronix. The preparation involved the following steps: First, the FTO glass substrates were cleaned using the procedure described in the substrate preparation section. The ready-to-use Pt solution was then applied to the cleaned FTO substrates using a spin-coating technique. Fifty microlitres of the Pt solution was spin-coated onto the FTO substrate at 1500 rpm for 10 seconds, followed by annealing at 450 °C for 15 minutes to form a uniform platinum layer on the FTO glass^28^.

### Modified TPT configuration and DSSC assembly

In the fabrication of the $\hbox {TiO}_2$ photoanodes, each FTO glass substrate was cleaned, UV-treated, and subjected to a $\hbox {TiCl}_4$ pre-treatment at 80 °C, followed by annealing at 450 °C. A screen-printing technique was used to apply the first $\hbox {TiO}_2$ layer, confining the active area to 0.25 $\hbox {cm}^2$, followed by annealing at 450 °C. The resulting mesoporous $\hbox {TiO}_2$ layer thickness was around 15 µm. This cycle was repeated for each subsequent layer. For the P25 layer, graphene nanopowder was incorporated at concentrations of 0%, 0.05%, 0.1%, and 0.2%. After the final layer, a $\hbox {TiCl}_4$ post-treatment and final annealing at 450 °C were performed to ensure a homogeneous and adherent multi-layered photoanode.

The CEs were prepared from FTO glass with pre-drilled holes. Following the cleaning protocol, the CEs were coated with 50 $\mu$L of Pt nanoparticle solution via spin-coating at 1500 rpm for 10 seconds, with a second application after 5 minutes. Post-coating, the electrodes were annealed at 450 °C. The photoanode samples were immersed in a 0.0002 M N719 dye solution in ethanol for 24 hours. Excess dye was removed with ethanol. A 60 $\mu$m polymer spacer was placed around the photoanode’s active area, and the heated CE was pressed onto the dye-coated photoanode. The electrolyte solution was injected through the pre-drilled holes to complete the assembly.

### Electrical characterization

Electrochemical Impedance Spectroscopy (EIS) investigated the charge dynamics within the DSSCs, assessing the impact of structural modifications on charge transfer resistance and recombination rates. J-V (current density-voltage) curve analysis measured the electrical performance of the DSSCs, deriving key performance indicators such as $\hbox {J}_{SC}$, open-circuit voltage ($\hbox {V}_{OC}$), fill factor (FF), and overall PCE.

### Optical characterization

Ultraviolet-visible (UV-Vis) spectroscopy evaluated the optical properties, focusing on light absorption efficiencies and bandgap shifts induced by graphene doping. This characterisation is essential for understanding the enhancement in the photoanodes’ light-harvesting capabilities, directly impacting the DSSCs’ performance.

### Structural and compositional analysis

Field Emission Scanning Electron Microscopy (FESEM) provided high-resolution visualisations of the surface morphology and cross-sectional architecture, verifying the uniformity and nanostructural integrity of the graphene-doped $\hbox {TiO}_2$ layers. Energy-dispersive X-ray spectroscopy (EDS) confirmed the elemental composition, ensuring successful graphene integration within the $\hbox {TiO}_2$ matrix. Raman spectroscopy offered insights into crystalline structure alterations and graphene integration, identifying key peaks corresponding to anatase $\hbox {TiO}_2$ and graphene and assessing the degree of disorder through the ID/IG ratio, which indicates the level of defects in the graphene structure. X-ray diffraction (XRD) identified the crystalline structures of $\hbox {TiO}_2$ phases, confirming the presence of anatase $\hbox {TiO}_2$, which is crucial for photocatalytic activity. This analysis helps understand the structural integrity and phase composition of the graphene-doped $\hbox {TiO}_2$ layers.

## Results and discussion

### Electrical characterization

#### J-V characteristics

To evaluate the performance of the DSSCs, we conducted current-voltage (J-V) measurements under both front and back illumination conditions (AM 1.5 G) solar mass over 10 days. This analysis provides critical insights into the cells’ PCE, $\hbox {J}_{SC}$, $\hbox {V}_{OC}$, and FF. Tracking these parameters over time allows us to assess the stability and efficiency improvements imparted by different graphene concentrations in the $\hbox {TiO}_2$ photoanodes. The J-V characteristics will enable us to compare the initial performance with the subsequent changes, highlighting the effect of graphene doping on the overall cell efficiency.

**TPT Configuration:** The performance of DSSCs with the TPT configuration was evaluated under both front and back illumination. The data is presented in Supporting Information Table 1 and Supporting Information Table 2, respectively. On day 0, the test was conducted 1-3 hours after electrolyte injection, with subsequent measurements taken on the following days. The results include initial observed and calculated data using an exponential equation to estimate values for intermediate days.

**Fig. 1:**
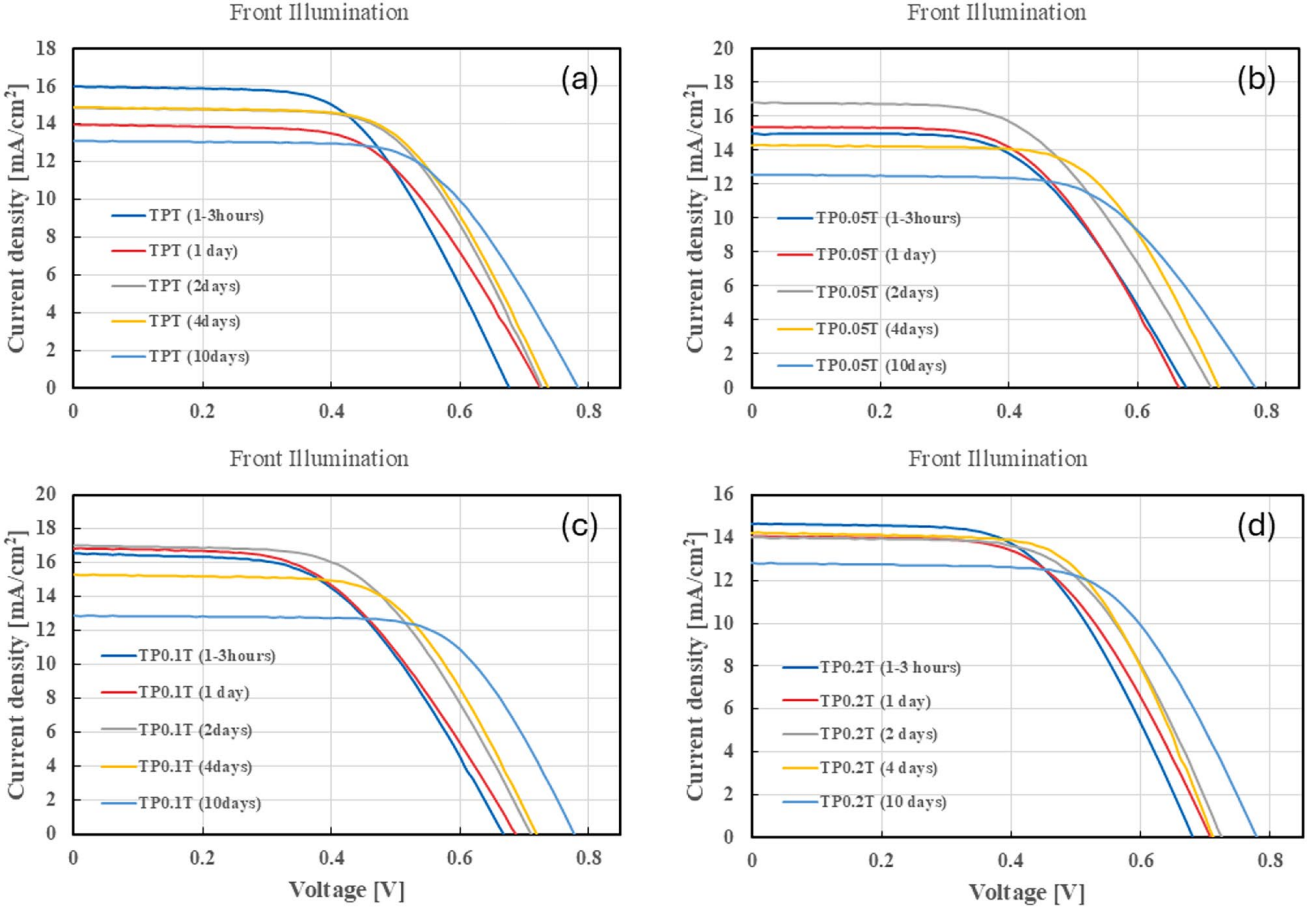
J-V curves for DSSCs under front illumination conditions over 10 days. The subfigures show the performance of different configurations: (**a**) TPT, (**b**) $\hbox {TP}_{0.05}$T, (**c**) $\hbox {TP}_{0.1}$T, and (**d**) $\hbox {TP}_{0.2}$T. These graphs illustrate the impact of varying graphene concentrations on the stability and efficiency of the cells under consistent front illumination.

Under front illumination, Supporting Information Table 1, the highest PCE was observed on day 4, reaching 6.71%. This high efficiency is attributed to the relatively stable values of $\hbox {J}_{SC}$ and $\hbox {V}_{OC}$, with $\hbox {J}_{SC}$ remaining above 14 mA/$\hbox {cm}^2$ and $\hbox {V}_{OC}$ around 0.74 V. The FF remained consistent at approximately 0.61. The maximum current density ($\hbox {J}_{max}$) also showed stability, with values close to 13 mA/$\hbox {cm}^2$. The high $R^2$ values for $\hbox {V}_{OC}$ (0.8911) and $\hbox {V}_{max}$ (0.8345) indicate a strong fit of the exponential model, suggesting the robustness of these parameters over the testing period. Figure 1a illustrates the J-V curves for the TPT configuration under front illumination. However, a constant degradation was observed after day 5, with the $\hbox {J}_{SC}$ slowly declining until 13.13 mA/$\hbox {cm}^2$ with a continuous rise in $\hbox {V}_{OC}$ to nearly 0.8 V at day 10. The increase in $\hbox {V}_{OC}$ DSSCs over time can be attributed to several factors, including structural optimisations^29^, interface modifications^30^, and the use of specific materials that reduce charge recombination^31^. In some solar cell technologies, such as perovskite solar cells, aging of precursor solutions can lead to a more uniform and smooth film formation, which reduces interfacial gaps and trap state density. This results in an increase in $\hbox {V}_{OC}$ over time, although this specific mechanism is not directly applicable to DSSCs, it highlights the importance of material processing in $\hbox {V}_{OC}$ enhancement^32^. Furthermore, The decline of the data could be from a build-up of resistance in the active area, where the electrolyte influences resistance and performance by affecting ionic mobility, recombination kinetics, and interfacial charge processes within the $\hbox {TiO}_2$ layer^33,34^.

**Fig. 2:**
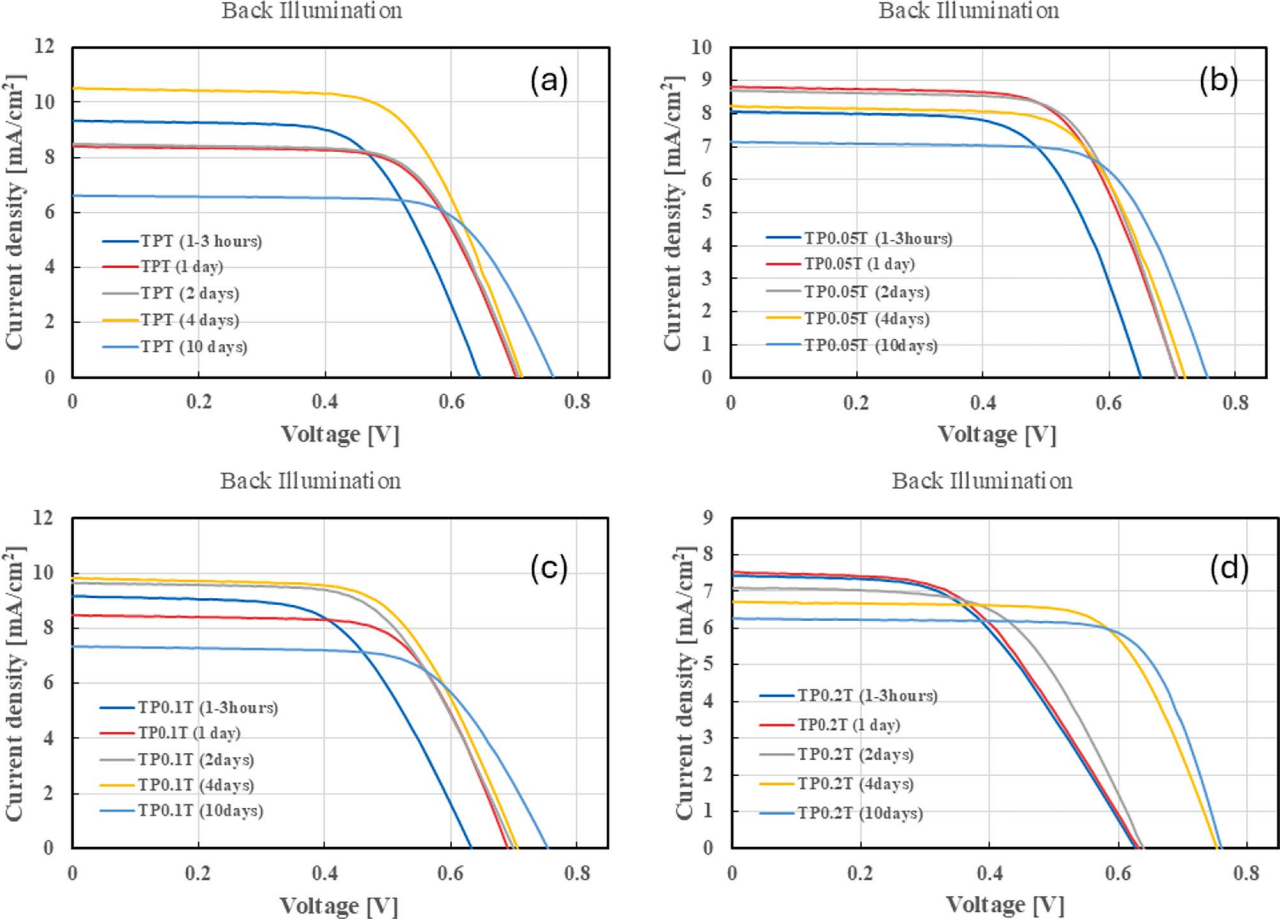
J-V curves for DSSCs under back illumination conditions over 10 days. The subfigures illustrate the performance variations of different configurations: (**a**) TPT, (**b**) $\hbox {TP}_{0.05}$T, (**c**) $\hbox {TP}_{0.1}$T, and (**d**) $\hbox {TP}_{0.2}$T. These curves highlight the impact of graphene doping on cell efficiency from the rear side of the panels, which is essential for understanding performance under less direct light conditions.

In contrast, under back illumination, as shown in Supporting Information Table 2, the PCE peaked earlier, on day 4, at 4.86%. The $\hbox {J}_{SC}$ values were lower than front illumination, peaking at 10.49 mA/$\hbox {cm}^2$, consistent with the expected lower efficiency under back illumination. The $\hbox {V}_{OC}$ values were slightly lower, peaking at 0.76 V. The $R^2$ values for $\hbox {J}_{SC}$ (0.3836) and $\hbox {J}_{max}$ (0.3803) indicate moderate fits, suggesting more variability and potential areas for improvement in the back illumination setup. Figure 2a shows the J-V curves for the TPT configuration under back illumination. Moreover, similar to the front illumination, the back illumination faced a decline in $\hbox {J}_{SC}$, suggesting steady and constant degradation after day 5.

${\textbf {TP}}_{0.05}$**T Configuration:** For the $\hbox {TP}_{0.05}$T configuration, the results under front illumination are shown in the Supporting Information Table 3. The peak PCE of 6.50% was observed on day 2, with a corresponding $\hbox {J}_{SC}$ of 16.80 mA/$\hbox {cm}^2$ and $\hbox {V}_{OC}$ of 0.72 V. The FF was relatively high at 0.54, and the $\hbox {J}_{max}$ was 14.44 mA/$\hbox {cm}^2$. This performance indicates stability enhancement after introducing graphene into the $\hbox {TiO}_2$ matrix^35^. The $R^2$ values for $\hbox {J}_{SC}$ (0.7662) and $\hbox {V}_{OC}$ (0.9401) suggest a good fit, indicating consistent performance over time. Figure 1b displays the J-V curves for the $\hbox {TP}_{0.05}$T configuration under front illumination. The $\hbox {J}_{SC}$ and PCE experienced degradation after day 5, similar to the TPT configuration, suggesting that the graphene percentage is not the optimum quantity needed for further stabilisation.

Under back illumination, as presented in Supporting Information Table 4, the peak PCE was 4.20% on day 2, with the $\hbox {J}_{SC}$ reaching 8.71 mA/$\hbox {cm}^2$ and the $\hbox {V}_{OC}$ at 0.71 V. The $R^2$ values for $\hbox {J}_{SC}$ (0.775) and $\hbox {J}_{max}$ (0.7302) indicate a better fit compared to the TPT configuration, suggesting that the $\hbox {TP}_{0.05}$T configuration may offer more stable performance under back illumination conditions. Figure 2b shows the J-V curves for the $\hbox {TP}_{0.05}$T configuration under back illumination. The cell remained relatively stable for a couple more days until it started to degrade after day 5.

${{\textbf {TP}}}_{0.1}$**T Configuration:** In the $\hbox {TP}_{0.1}$T configuration, front illumination results are displayed in Supporting Information Table 5. The highest PCE of 6.73% was observed on day 2, with $\hbox {J}_{SC}$ of 17.04 mA/$\hbox {cm}^2$ and $\hbox {V}_{OC}$ of 0.71 V. The FF was 0.56, and $\hbox {J}_{max}$ was 14.64 mA/$\hbox {cm}^2$. The $R^2$ values for $\hbox {J}_{SC}$ (0.9459) and $\hbox {V}_{OC}$ (0.9794) are exceptionally high, indicating very reliable performance metrics for these parameters. Figure 1c shows the J-V curves for the $\hbox {TP}_{0.1}$T configuration under front illumination. Although the $\hbox {J}_{SC}$ began to decline after the 3rd to 5th day, the PCE remained stable until day 10 with minimal degradation, suggesting that the 0.1wt% graphene into the $\hbox {TiO}_2$ greatly enhanced the stability of the cell.

For back illumination, as presented in Supporting Information Table 6, the peak PCE was 4.36% on day 4, with $\hbox {J}_{SC}$ at 9.84 mA/$\hbox {cm}^2$ and $\hbox {V}_{OC}$ at 0.71 V. The $R^2$ values for $\hbox {J}_{SC}$ (0.502) and $\hbox {J}_{max}$ (0.4527) indicate a moderate fit, suggesting areas for optimisation in the back illumination performance. The J-V curves for the $\hbox {TP}_{0.1}$T configuration under back illumination are shown in Figure 2c. The PCE remained relatively above 4% from the 2nd to the 6th day of testing, suggesting a better performance at 0.1wt% graphene than 0.05wt%.

${\textbf {TP}}_{0.2}$**T Configuration:** The $\hbox {TP}_{0.2}$T configuration under front illumination is summarised in Supporting Information Table 7. A peak PCE of 6.29% was achieved on day 4, with $\hbox {J}_{SC}$ of 14.24 mA/$\hbox {cm}^2$ and $\hbox {V}_{OC}$ of 0.72 V. The FF was 0.61, and $\hbox {J}_{max}$ was 12.84 mA/$\hbox {cm}^2$. The $R^2$ values for $\hbox {J}_{SC}$ (0.8682) and $\hbox {V}_{OC}$ (0.9263) indicate a firm fit, demonstrating stable performance over time. Figure 1d illustrates the J-V curves for the $\hbox {TP}_{0.2}$T configuration under front illumination. This configuration shows that a higher graphene concentration does not necessarily lead to improved performance, as the $\hbox {J}_{SC}$ and $\hbox {J}_{max}$ declined from the previous concentrations, supporting the finding that 0.1wt% is the optimum concentration for the TPT configuration.

Under back illumination, as detailed in Supporting Information Table 8, the peak PCE was 3.52% on day 6, with $\hbox {J}_{SC}$ at 6.56 mA/$\hbox {cm}^2$ and $\hbox {V}_{OC}$ at 0.76 V. The $R^2$ values for $\hbox {J}_{SC}$ (0.9297) and $\hbox {J}_{max}$ (0.9211) suggest a very good fit, indicating that the $\hbox {TP}_{0.2}$T configuration performs consistently under back illumination conditions. Figure 2d shows the J-V curves for the $\hbox {TP}_{0.2}$T configuration under back illumination.

The data highlights the enhanced performance and stability of bifacial DSSCs with varying concentrations of graphene under both front and back illumination. The $\hbox {TP}_{0.1}$T configuration showed the most promising results with the highest $R^2$ values, suggesting its suitability for further development. The consistent performance under back illumination also indicates the potential for practical applications in environments where both sides of the cell can be exposed to light.

**Fig. 3:**
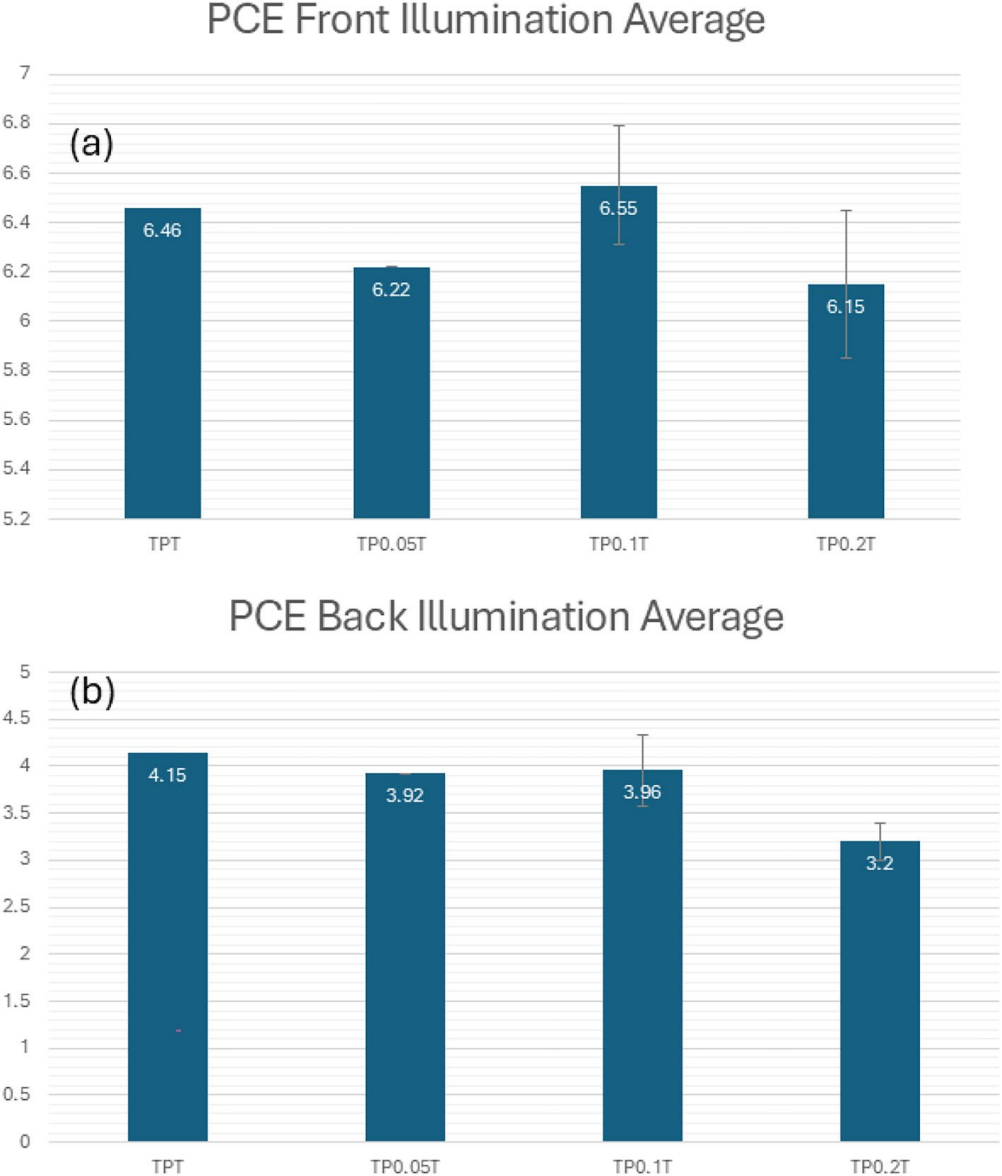
Time-averaged power conversion efficiency (PCE) of TPT and graphene-doped photoanodes over the full 10-day stability test period under (**a**) front and (**b**) back illumination. Bars represent the mean ± SD (n = 2 pseudo-replicates: front/back measurements of the same device) taken across all measurement days.

Figure 3 condenses the complete ten-day data set by displaying the arithmetic mean and standard deviation of daily PCE values for each sample. Under front illumination [panel (a)], the TP$_{0.1}$T device attains the highest time-averaged efficiency of 6.55% ± 0.32%, slightly surpassing the undoped TPT control (6.46% ± 0.24%) and the TP$_{0.05}$T (6.22% ± 0.30%) and TP$_{0.2}$T (6.15% ± 0.23%) photoanodes. For back illumination [panel (b)], the ranking is led by TPT at 4.15% ± 0.38%, followed by TP$_{0.1}$T (3.96% ± 0.28%), TP$_{0.05}$T (3.92% ± 0.20%), and TP$_{0.2}$T (3.20% ± 0.45%). These time-averaged results confirm that a 0.1% graphene loading not only yields the highest peak efficiency but also sustains superior front-side performance throughout the entire stability test while maintaining competitive back-side operation.

By the 10th day of the experiment, as shown in Table 1, the results showed a high stability regarding $\hbox {TP}_{0.2}$T that can be attributed to the high concentration of graphene in the $\hbox {TiO}_{2}$ matrix. Similarly, but with higher PCE can be shown in $\hbox {TP}_{0.1}$T. TPT and $\hbox {TP}_{0.05}$T shown much less stability with higher degradation effect. Summing the front and back illumination efficiencies, the following total PCE values at the 10th day were observed:

- TPT: 9.96% (6.40% + 3.56%)
- $$\hbox {TP}_{0.05}$$T: 9.84% (6.03% + 3.81%)
- $$\hbox {TP}_{0.1}$$T: 10.31% (6.68% + 3.63%)
- $$\hbox {TP}_{0.2}$$T: 9.81% (6.29% + 3.52%)

**Table 1:** The front and back illumination of the standard and graphene-enhanced TPT configurations devices by the 10th day.

Configuration	$\hbox {J}_{SC}$ [mA/$\hbox {cm}^2$]	$\hbox {V}_{OC}$ [V]	FF	PCE [%]	$\hbox {J}_{max}$ [mA/$\hbox {cm}^2$]	$\hbox {V}_{max}$ [V]
TPT (Front)	13.13	0.79	0.62	6.40	11.85	0.54
TPT (Back)	6.64	0.77	0.70	3.56	6.14	0.58
$\hbox {TP}_{0.05}$T (Front)	12.60	0.79	0.61	6.03	11.16	0.54
$\hbox {TP}_{0.05}$T (Back)	7.16	0.76	0.70	3.81	6.56	0.58
$\hbox {TP}_{0.1}$T (Front)	12.88	0.78	0.66	6.68	11.72	0.57
$\hbox {TP}_{0.1}$T (Back)	7.33	0.76	0.65	3.63	6.60	0.55
$\hbox {TP}_{0.2}$T (Front)	12.84	0.78	0.63	6.29	11.44	0.55
$\hbox {TP}_{0.2}$T (Back)	6.25	0.77	0.73	3.52	5.87	0.60

These findings reinforce the notion that strategic graphene doping can significantly influence the efficacy of bifacial DSSCs, optimising their operational capabilities in varied lighting conditions.

Due to the inherent design of DSSCs, which favours front illumination, the PCE tends to be higher when light is absorbed from the front. This is primarily because the dye molecules, crucial for light harvesting, are more effectively positioned to capture and utilise light entering through the front of the cell^36^. To quantify the performance difference between front and back illumination, we calculate the bifacial factor (BFF) using the formula:

1 $$\begin{aligned} \text {BFF} = \frac{\text {PCE}_{\text {back}}}{\text {PCE}_{\text {front}}}. \end{aligned}$$

where $\text {PCE}_{\text {back}}$ and $\text {PCE}_{\text {front}}$ represent the power conversion efficiencies under the back and front illumination, respectively. This metric helps understand the cell’s efficiency from both sides and highlights its potential for varied lighting conditions, particularly in applications where both sides may receive light^37^.

The BFF ratios presented in Table 2 reveal significant insights into the performance of DSSCs under different graphene doping conditions. TPT configurations exhibit the highest BFF ratio, peaking at approximately 0.72, indicating a substantial drop in efficiency when transitioning from front-to-back illumination. This trend suggests that while efficient under direct illumination, the standard configuration loses considerable performance when exposed from the rear. The $\hbox {TP}_{0.05}$T configuration shows a higher maximum BFF ratio of around 0.71, implying a slightly better balance between front and back illumination efficiencies, potentially due to the light scattering effects of the graphene. Notably, the $\hbox {TP}_{0.1}$T configuration achieves a moderately high ratio but demonstrates more consistency across different measurements, suggesting an optimal graphene concentration for maintaining balance in bifacial applications. Conversely, the $\hbox {TP}_{0.2}$T configuration consistently shows lower BFF ratios, indicating diminishing returns at higher graphene concentrations, likely due to increased optical density that impedes light penetration to the rear side of the cells. These findings underscore the importance of optimising graphene concentration in the photoanode to achieve a desirable balance between high efficiency and stable bifacial performance.

**Table 2:** Bifaciality factor ratios for different graphene concentrations.

Day	TPT BFF ratio	$\hbox {TP}_{0.05}$T Ratio	$\hbox {TP}_{0.1}$T BFF Ratio	$\hbox {TP}_{0.2}$T BFF Ratio
1-3 hours	0.619488	0.606183	0.579870	0.424344
Day 1	0.682989	0.713426	0.663732	0.432314
Day 2	0.614823	0.645941	0.619750	0.437583
Day 3	0.682447	0.635098	0.630507	0.492020
Day 4	0.724470	0.606138	0.648635	0.558154
Day 5	0.693359	0.610323	0.629715	0.558347
Day 6	0.663585	0.614538	0.611347	0.558541
Day 7	0.635089	0.618781	0.593515	0.558735
Day 8	0.607817	0.623054	0.576203	0.558929
Day 9	0.581716	0.627356	0.559396	0.559123
Day 10	0.556736	0.631688	0.543079	0.559317

#### Analysis of PCE trends over time

The PCE trends were analysed over 10 days to understand the performance and stability of the DSSCs with various graphene concentrations. By plotting the PCE values over time for both front and back illumination, we can observe the peak performance days and the overall stability of the cells. This trend analysis is crucial for identifying the optimal graphene concentration that offers the best balance between initial performance and stability, providing valuable insights into the practical application of these cells in real-world conditions.

For the TPT configuration, the highest front illumination PCE was observed on day 4, reaching 6.71%, while the back illumination PCE peaked on the same day at 4.86%. The $\hbox {TP}_{0.05}$T configuration showed its highest front illumination PCE on day 2 at 6.50%, with the back illumination peaking at 4.20% on the same day. The $\hbox {TP}_{0.1}$T configuration achieved its highest front illumination PCE of 6.73% on day 2, with the back illumination peaking on day 4 at 4.36%. Lastly, the $\hbox {TP}_{0.2}$T configuration reached its highest front illumination PCE of 6.29% on day 4, while the back illumination peaked on day 6 at 3.52%.

**Fig. 4:**
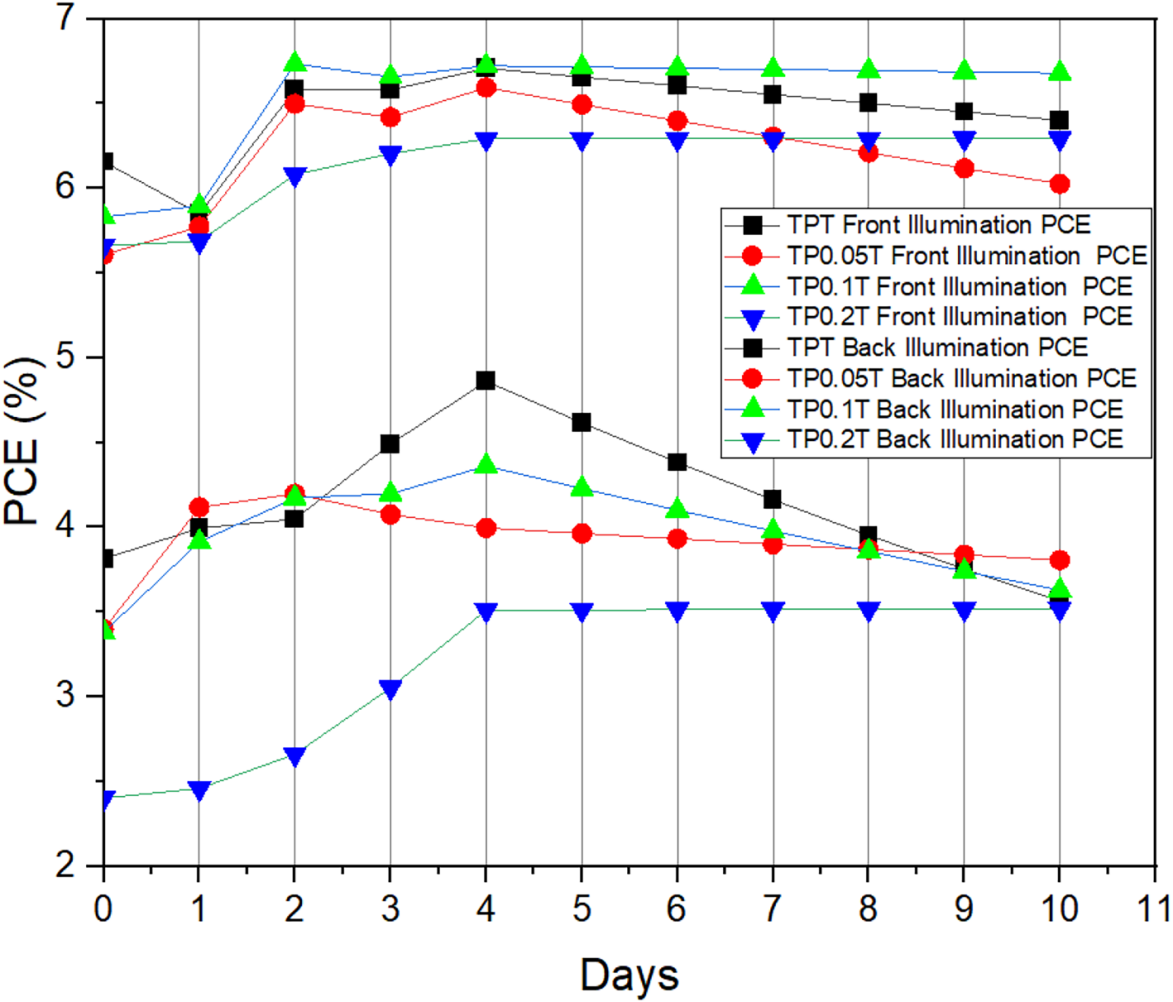
Power conversion efficiency (PCE) trends over 10 days for DSSCs with varying graphene concentrations under front and back illumination.

Figure 4 effectively summarises the performance trends, showing each configuration’s relative stability and efficiency over time. The consistent performance under back illumination indicates potential for practical applications in environments where both sides of the cell can be exposed to light.

#### Electrochemical impedance spectroscopy (EIS)

**Fig. 5:**
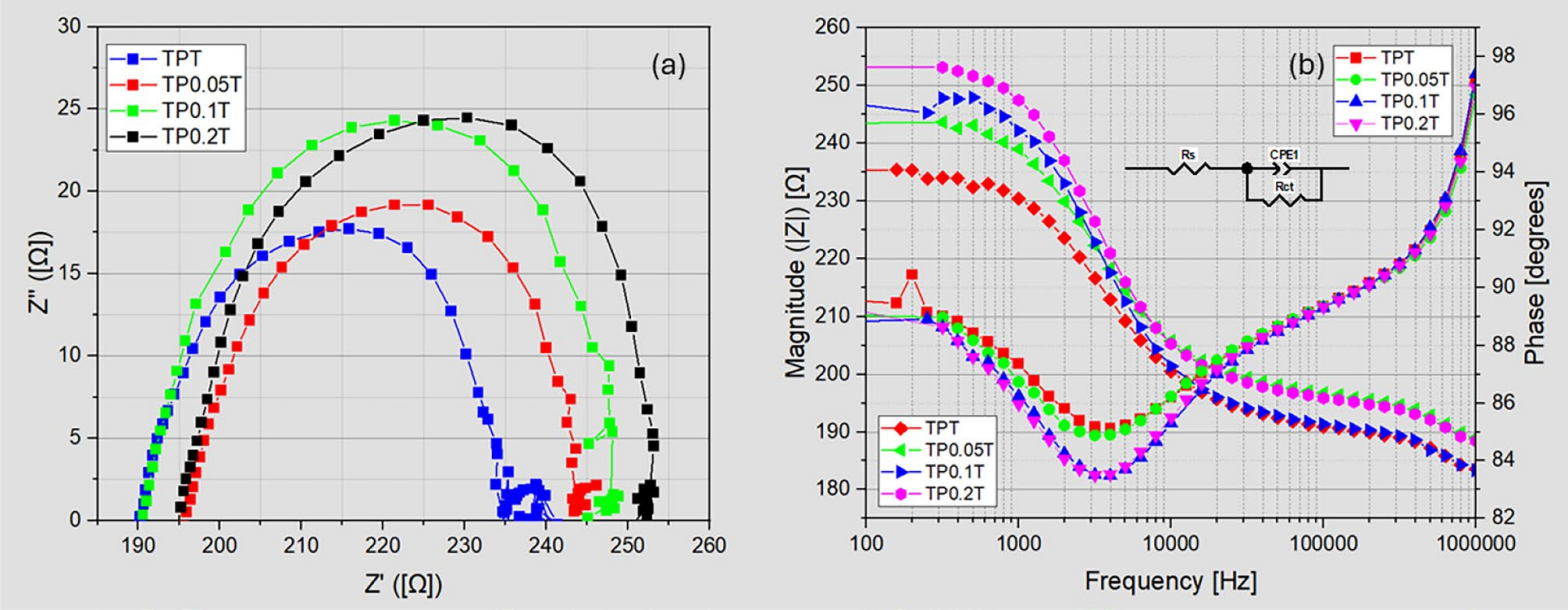
EIS Nyquist and Bode plots for enhanced TPT with varying graphene concentrations illustrating charge transfer and diffusion characteristics. Panel (**a**) shows Nyquist plots, and panel (**b**) shows Bode plots.

The electrochemical impedance spectroscopy (EIS) analysis revealed that incorporating graphene into $\hbox {TiO}_2$ photoanodes significantly enhances the electrochemical performance of DSSCs^38^. As illustrated in the Nyquist plots (Figure 5a), the semicircle diameters, which correlate with the charge transfer resistance (Rct), increase with higher graphene content, indicating that while graphene enhances electronic pathways within the $\hbox {TiO}_2$ matrix, it also introduces interfacial complexities that can hinder charge transfer. The Bode plots (Figure 5b) further support this by showing changes in the phase angle and impedance magnitude, highlighting improved charge separation and electron lifetime, especially in the $\hbox {TP}_{0.1}$T sample.

Table 3 provides a comprehensive overview of the electrochemical parameters for the different DSSC configurations. The Warburg impedance parameters (Wo-R, Wo-T, and Wo-P) provide insights into the ionic diffusion characteristics within the cells. Lower Wo-R values in the $\hbox {TP}_{0.1}$T and $\hbox {TP}_{0.2}$T samples suggest improved ionic diffusion, crucial for efficient dye regeneration and overall device performance. Specifically, the Wo-R value for $\hbox {TP}_{0.1}$T is 1.50 $\Omega \,\text {S}^{-0.5}$, significantly lower than the 9.53 $\Omega \,\text {S}^{-0.5}$ observed in the undoped TPT sample. This improvement in ionic transport is corroborated by the enhanced fill factors observed in the J–V characteristics of the graphene-doped DSSCs.

**Table 3:** Electrochemical parameters from EIS for DSSCs with different graphene concentrations, showing series resistance, charge transfer resistance, electron lifetime, and Warburg parameters.

Sample	R_S_ ($\Omega$)	R_CT_ ($\Omega$)	$\tau _e$ ($\mu$s)	CPE (nF)	$\omega _{\text {max}}$ (Hz)	Wo-R ($\Omega$)	Wo-T (ns)	Wo-P
TPT	190.4	45.31	31.8	179.35	4.9142	9.53m	2.28	432.88m
$\hbox {TP}_{0.05}$T	196.2	46.98	50.3	120.62	5.0978	2.77m	0.700	442.19m
$\hbox {TP}_{0.1}$T	190.8	57.44	63.4	19.20	24.1400	1.50m	0.359	450.19m
$\hbox {TP}_{0.2}$T	195.3	59.07	50.3	23.90	25.2220	1.08m	0.237	446.05m

Building on the Warburg analysis, charge-carrier dynamics can be assessed by the electron lifetimes $\tau _e$ and corresponding recombination rates

$$\begin{aligned} k_\textrm{rec} = \frac{1}{\tau _e}. \end{aligned}$$

From Table 3, the undoped TPT sample exhibits $\tau _e = {31.8}{\upmu \textrm{s}}$, whereas $\hbox {TP}_{0.05}$T, $\hbox {TP}_{0.1}$T, and $\hbox {TP}_{0.2}$T show extended lifetimes of 50.3${\upmu \textrm{s}}$, 63.4${\upmu \textrm{s}}$, and 50.3${\upmu \textrm{s}}$, respectively. Converting these to recombination rates yields:

2 $$\begin{aligned} k_\textrm{rec} = \frac{1}{\tau _e} \quad \Longrightarrow \quad {\left\{ \begin{array}{ll} k_\textrm{rec}^\textrm{TPT} \approx 3.14 \times 10^{4}\,\text {s}^{-1},\\ k_\textrm{rec}^\mathrm{TP_{0.05}T} \approx 1.99 \times 10^{4}\,\text {s}^{-1},\\ k_\textrm{rec}^\mathrm{TP_{0.1}T} \approx 1.58 \times 10^{4}\,\text {s}^{-1},\\ k_\textrm{rec}^\mathrm{TP_{0.2}T} \approx 1.99 \times 10^{4}\,\text {s}^{-1}. \end{array}\right. } \end{aligned}$$

Notably, the $\hbox {TP}_{0.1}$T sample exhibits the longest $\tau _e$ and lowest $k_\textrm{rec}$, corresponding to a $\sim \!50\%$ reduction in recombination rate compared to undoped TPT. This suppression of carrier recombination underpins the enhanced charge-transfer efficiency. It aligns with the superior PCE and fill factor observed in the J–V characteristics of the $\hbox {TP}_{0.1}$T DSSC.

Integrating graphene into $\hbox {TiO}_2$ photoanodes enhances electronic and ionic transport properties, improving device performance. The optimal concentration of graphene appears to be around 0.1wt%, balancing the benefits of enhanced electron mobility and reduced recombination rates with the complexities introduced at higher doping levels. These findings underscore the potential of graphene-doped $\hbox {TiO}_2$ photoanodes in advancing the efficiency and stability of DSSCs, making them more viable for practical applications^39^.

### Optical characterisation: UV-vis absorption spectra and tauc plots

UV-vis absorption spectroscopy was performed to evaluate the optical properties of the graphene-doped $\hbox {TiO}_2$ samples, and the data were analysed using Tauc plots. UV-vis absorption spectra reveal how adding graphene influences the light absorption capabilities of $\hbox {TiO}_2$, particularly in the visible region. Derived Tauc plots allow for the estimation of the samples’ bandgap energies, which is critical for understanding enhancements in photocatalytic activity and overall cell performance. This optical characterisation helps assess how graphene doping modifies the electronic structure of $\hbox {TiO}_2$, contributing to improved efficiency in DSSCs^40^.

**Fig. 6:**
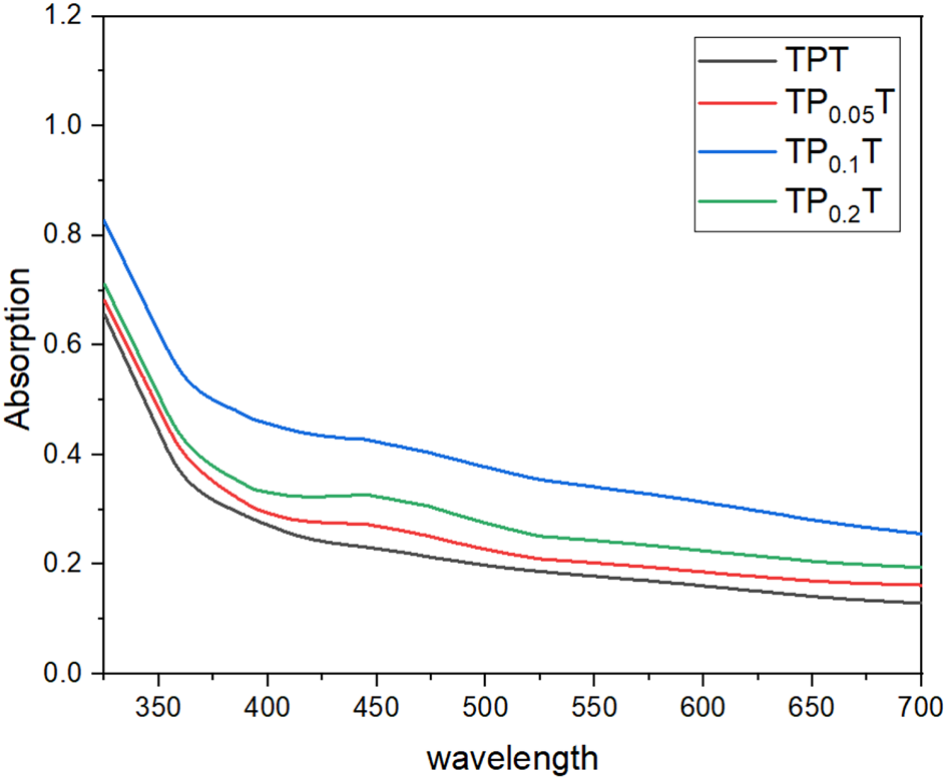
UV-Vis absorption spectra of undoped and graphene-doped $\hbox {TiO}_{2}$ samples indicate the effect of graphene concentration on $\hbox {TiO}_{2}$’s light absorption properties.

The UV-vis absorption spectra Figure 6 of the graphene-doped $\hbox {TiO}_2$ samples, measured without dye absorption, illustrates the impact of graphene on the photoanodes’ optical properties. For the TPT (undoped $\hbox {TiO}_2$) sample, the baseline absorption spectrum exhibits typical $\hbox {TiO}_2$ characteristics with a prominent absorption edge around 380 nm, indicative of its intrinsic bandgap. The $\hbox {TP}_{0.05}$T sample shows a slight increase in absorption across the spectrum, suggesting enhanced light-harvesting capabilities due to the presence of graphene. The $\hbox {TP}_{0.1}$T sample exhibits a significant increase in absorption in the visible region, indicating that higher concentrations of graphene further improve light absorption properties^41,42^. However, the $\hbox {TP}_{0.2}$T sample shows a moderate increase in absorption compared to $\hbox {TP}_{0.05}$T, but slightly lower than $\hbox {TP}_{0.1}$T, suggesting an optimal graphene concentration for maximising light absorption.

**Fig. 7:**
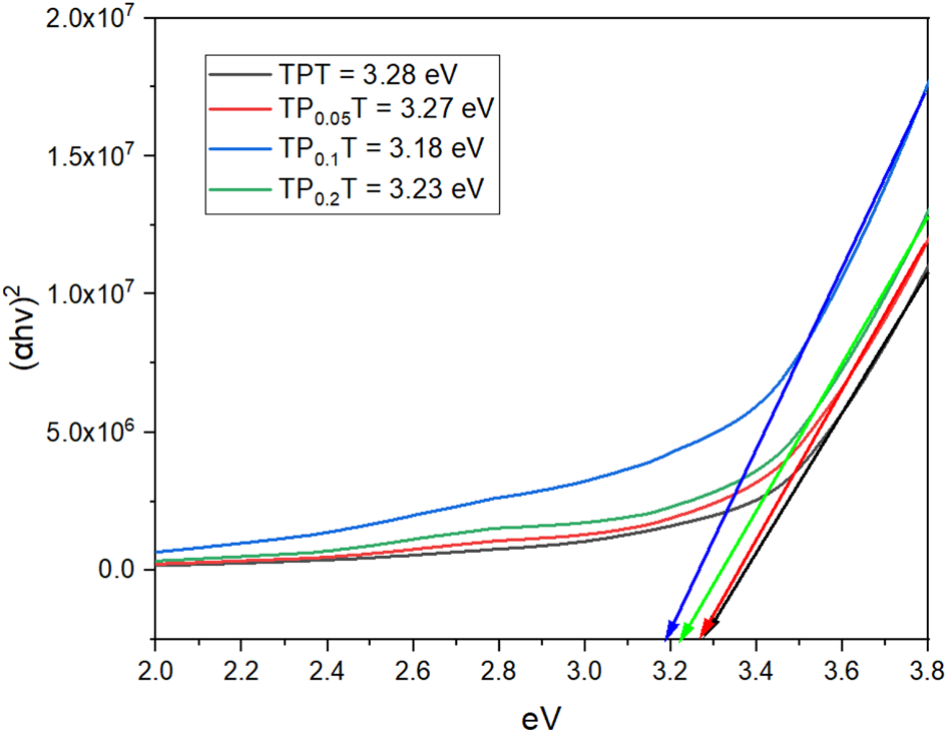
Tauc plots derived from UV-Vis data for undoped and graphene-doped $\hbox {TiO}_{2}$ samples showing bandgap energies that decrease with increasing graphene concentration.

The Tauc Plots, Figure 7, and Table 4 derived from the UV-vis absorption data provide insights into the optical bandgap energies of the samples. The TPT sample has an optical bandgap of approximately 3.28 eV, consistent with anatase $\hbox {TiO}_2$. Introducing 0.05% graphene ($\hbox {TP}_{0.05}$T) slightly decreases the bandgap to 3.27 eV, indicating minor alterations in the electronic structure. An increasingly further reduction to 3.18 eV is observed for the $\hbox {TP}_{0.1}$T sample, corresponding to the enhanced visible region absorption seen in the UV-vis spectra, suggesting significant modification of the $\hbox {TiO}_2$ band structure. The $\hbox {TP}_{0.2}$T sample’s bandgap slightly increases to 3.23 eV, indicating a saturation point where further graphene addition does not significantly enhance electronic properties and may even slightly decrease performance^43^. These findings confirm that optimal graphene doping can modulate the bandgap of $\hbox {TiO}_2$, improving light absorption and potential photocatalytic efficiency, which is crucial for optimising graphene concentration in $\hbox {TiO}_2$ photoanodes for DSSC applications.

**Table 4:** Estimated bandgap energies for photoanodes with various graphene concentrations.

Sample	Bandgap energy (eV)	Notes
TPT	3.28	Base material
$\hbox {TP}_{0.05}$T	3.27	0.05% graphene doping
$\hbox {TP}_{0.1}$T	3.18	0.1% graphene doping
$\hbox {TP}_{0.2}$T	3.23	0.2% graphene doping

### Structural and compositional analysis: FESEM, EDS, XRD, and Raman

To characterise the morphology and elemental composition of the graphene-doped $\hbox {TiO}_2$ photoanodes, we employed field emission scanning electron microscopy (FESEM) and energy-dispersive X-ray spectroscopy (EDS). The FESEM analysis provides detailed images of the surface structure, allowing us to observe the dispersion and integration of graphene within the $\hbox {TiO}_2$ matrix. EDS complements this by offering quantitative data on the elemental composition, confirming the presence and distribution of carbon (from graphene), oxygen, and titanium. These techniques help us understand how the structural properties of the photoanodes influence their performance in DSSCs.

#### The field emission scanning electron microscopy (FESEM)

**Fig. 8:**
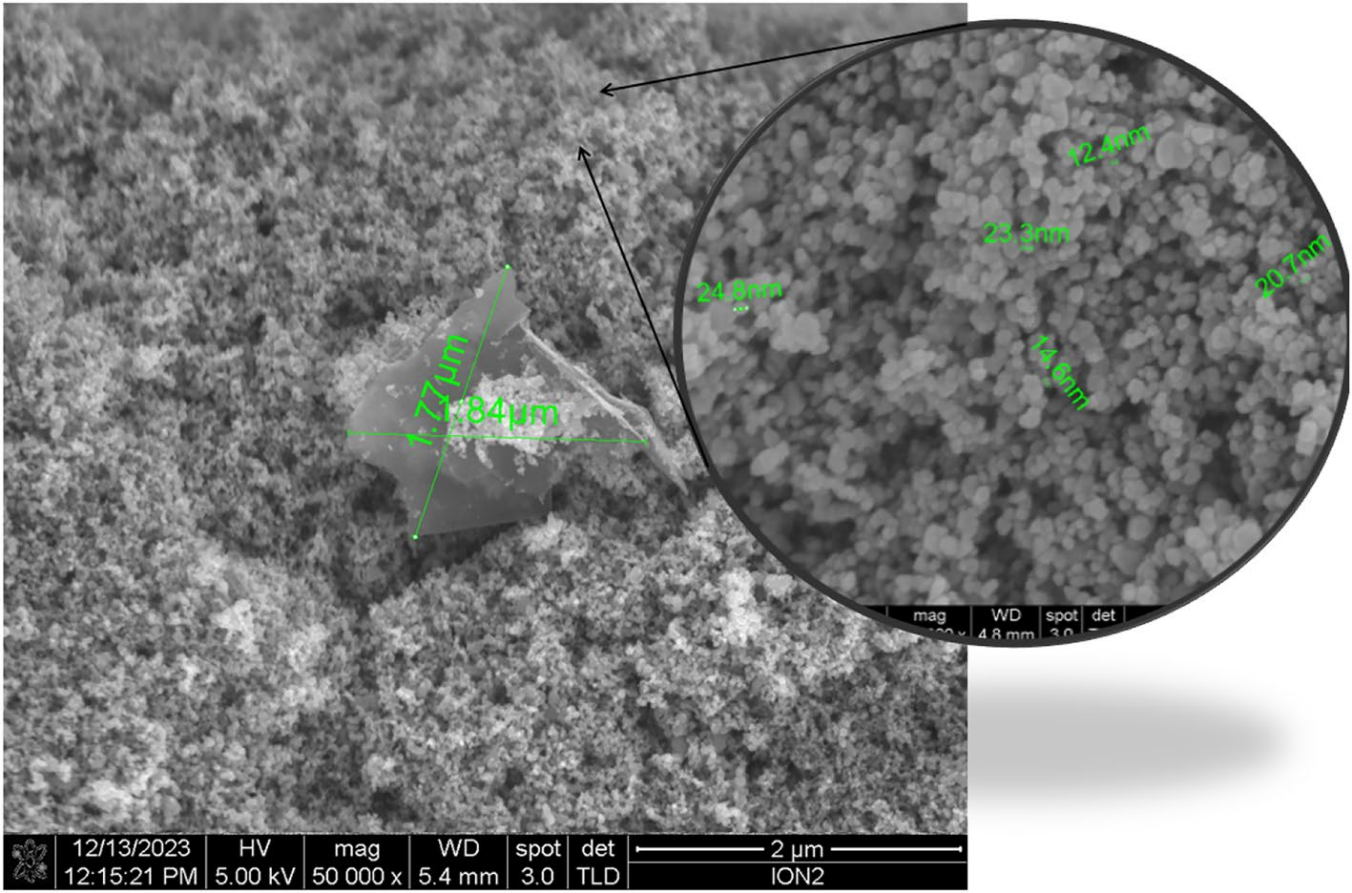
FESEM image of a graphene-doped $\hbox {TiO}_2$ photoanode for DSSCs highlighting distinct graphene flakes (approximately 1.77$\mu$m by 1.84$\mu$m).

Figure 8 showcases the surface morphology of the graphene-doped $\hbox {TiO}_2$ photoanode utilized in our DSSCs. A prominent feature is a distinct graphene nanosheet, measuring approximately 1.77$\upmu \textrm{m}$ by 1.84$\upmu \textrm{m}$, situated within the $\hbox {TiO}_2$ matrix. This flat, well-defined structure confirms successful integration of graphene flakes into the semiconductor. The surrounding $\hbox {TiO}_2$ appears densely packed, with a granular texture typical of nanoparticulate anatase. The contrast between the smooth geometry of the graphene and the granular $\hbox {TiO}_2$ highlights the heterogeneity introduced by doping, which is expected to improve electron transport and increase surface area for dye adsorption.

From the same FESEM images (see inset of Figure 8), primary $\hbox {TiO}_2$ particles were measured at 12.4nm, 14.6nm, 20.7nm, 23.3nm and 24.8nm, suggesting a broad size distribution. A quantitative analysis of $n=100$ particles using ImageJ yields an average diameter of 19.2nm ± 5.4nm (range: 12.4nm to 24.8nm). These SEM-derived grain sizes exceed the XRD-derived crystallite sizes (4nm to 6nm) obtained from Scherrer analysis (Table 6), indicating that each mesoporous $\hbox {TiO}_2$ grain comprises multiple smaller anatase domains.

#### Energy-dispersive X-ray spectroscopy (EDX)

**Fig. 9:**
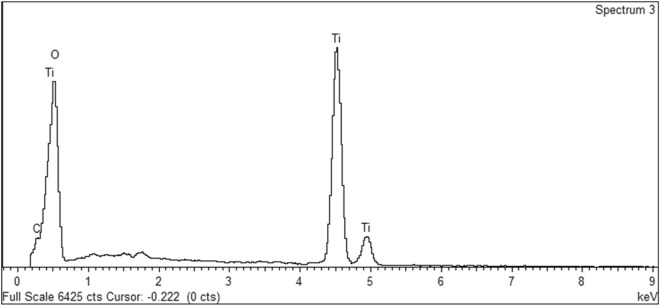
EDX spectrum of graphene-doped $\hbox {TiO}_2$ highlighting the peaks corresponding to carbon (C), oxygen (O), and titanium (Ti), indicative of the material’s composition.

The elemental composition of the graphene-doped $\hbox {TiO}_2$ photoanodes, pivotal to enhancing the performance of DSSCs, was determined through energy-dispersive X-ray spectroscopy (EDS). As elucidated in Figure 9 and quantitatively summarised in Table 5, the EDS analysis confirmed the presence of carbon (C), oxygen (O), and titanium (Ti) within the samples, with carbon signifying the successful doping of graphene into the $\hbox {TiO}_2$ matrix. The analysis yielded weight percentages of 5.88% for carbon, 55.45% for oxygen, and 38.67% for titanium, translating to atomic percentages of 10.29%, 72.77%, and 16.95%, respectively. These results verify the incorporation of graphene and underscore the stoichiometric balance of $\hbox {TiO}_2$ within the composite material. The specific ratios of graphene to $\hbox {TiO}_2$ offer insights into the doping level, providing a basis for understanding the observed enhancements in photocatalytic activity and photovoltaic performance of the DSSCs. To calculate the atomic weight% between C and Ti :o, we have used a simple equation of C = 10.3 %, Ti:O = 89.7 %, resulting in $\approx$ a 0.115 ratio, which is about 1 C atom for every 8.7 Ti:O atoms.

**Table 5:** Elemental composition of graphene-doped $\hbox {TiO}_2$ photoanodes as determined by EDS, showing weight and atomic percentages of carbon (C), oxygen (O), and titanium (Ti).

Element	Weight %	Atomic %
C K	5.88	10.29
O K	55.45	72.77
Ti K	38.67	16.95
**Totals**	100.00	-

#### X-ray diffraction (XRD) analysis

**Fig. 10:**
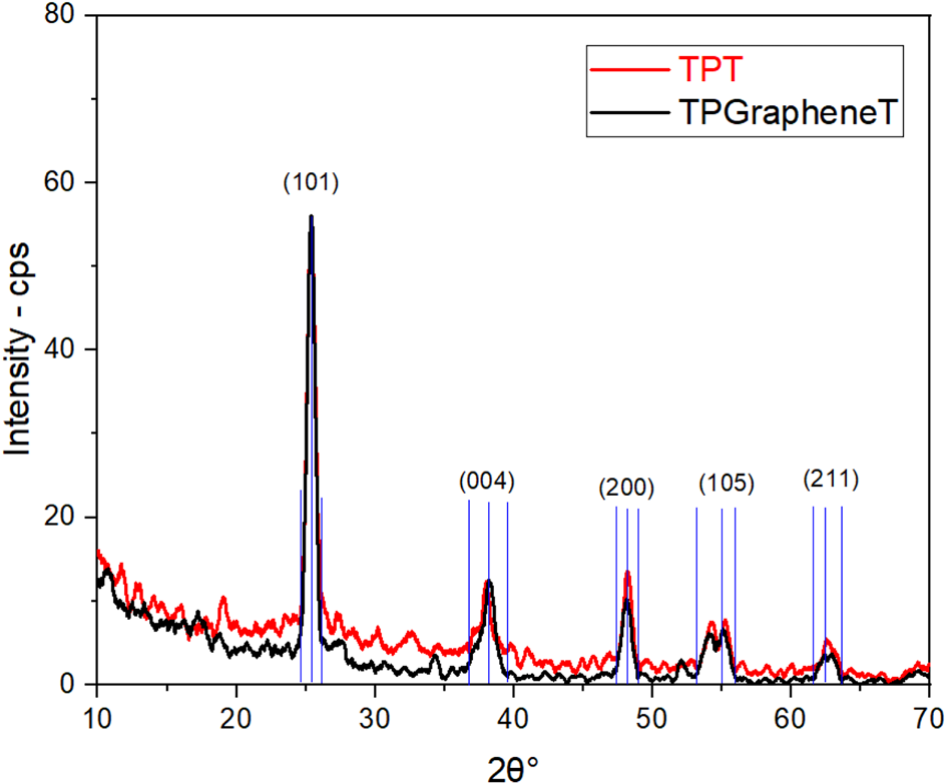
XRD patterns of TPT (pure $\hbox {TiO}_2$) and $\hbox {TP}_{graphene}$T (graphene-doped $\hbox {TiO}_2$) samples, showing diffraction peaks corresponding to different crystallographic planes of anatase $\hbox {TiO}_2$.

The crystalline structures of the $\hbox {TiO}_2$ phases in both pure $\hbox {TiO}_2$ (TPT) and graphene-doped $\hbox {TiO}_2$ ($\hbox {TP}_{graphene}$T) samples were characterised using X-ray diffraction (XRD). Figure 10 shows the XRD patterns for both samples, highlighting the diffraction peaks corresponding to different crystallographic planes of anatase $\hbox {TiO}_2$. The peaks are labelled according to their respective planes: (101), (004), (200), (105), and (211). The (101) peak, the most intense and characteristic peak of anatase $\hbox {TiO}_2$, appears around $2\theta = 25.3^\circ$, confirming the presence of the anatase phase in both samples. The other peaks observed at $2\theta = 37.8^\circ$ (004), $48.0^\circ$ (200), $53.9^\circ$ (105), and $62.7^\circ$ (211) are also consistent with the anatase phase, indicating that the crystalline structure of $\hbox {TiO}_2$ is maintained upon graphene doping. The intensity of the peaks for the TPT sample is higher than the $\hbox {TP}_{graphene}$T sample, suggesting that introducing graphene slightly reduces the crystallinity of $\hbox {TiO}_2$. This reduction in intensity is typical when additional materials like graphene are incorporated into the $\hbox {TiO}_2$ matrix, as they can introduce some degree of disorder or strain within the crystal structure. However, the preservation of the anatase peaks in the $\hbox {TP}_{graphene}$T sample indicates that the structural integrity of $\hbox {TiO}_2$ is primarily maintained even after doping with graphene. The XRD analysis confirms that the predominant phase in TPT and $\hbox {TP}_{graphene}$T samples is anatase $\hbox {TiO}_2$. The slight reduction in peak intensity for the graphene-doped sample suggests the successful incorporation of graphene without significantly disrupting the $\hbox {TiO}_2$ crystal structure.

To further analyse the crystallite sizes of the $\hbox {TP}_{graphene}$T sample, we have employed Scherrer analysis. $D$ (the diameter) of the photoanodes were extracted from the full-width at half-maximum () of five prominent anatase peaks using the following equation:

3 $$\begin{aligned} D = \frac{k\,\lambda }{\beta \,\cos \theta } \end{aligned}$$

Crystallite sizes $D$ of the $\hbox {TP}_{graphene}$T photoanodes were extracted from the full-width at half-maximum ($\beta$) of five prominent anatase peaks using the Debye-Scherrer equation (Eq. 3), where $k$ is the shape factor (0.90), $\lambda$ the X-ray wavelength (0.15?nm), and $\theta$ the Bragg angle. As shown in Table 6, the (101) reflection yields the largest crystallite size (6.00?nm), while higher-order reflections such as (004) and (105) give smaller sizes (2.95nm to 2.96nm). The average crystallite size over all five peaks is $4.06\pm 1.17$ nm, consistent with a nanocrystalline anatase network that supports efficient dye adsorption and electron transport.

**Table 6:** Crystallite sizes of $\hbox {TP}_{graphene}$T photoanodes calculated via the Scherrer equation.

Peak	${k}$	${\lambda }$ (nm)	${\beta }$ (rad)	${\theta }$ (rad)	${D}$ (nm)
(101)	0.90	0.15	0.03	0.49	6.00
(004)	0.90	0.15	0.05	0.11	2.96
(200)	0.90	0.15	0.03	0.09	4.98
(105)	0.90	0.15	0.05	0.06	2.95
(211)	0.90	0.15	0.03	0.03	4.41

#### Raman spectroscopy

Raman spectroscopy was utilised to investigate the structural properties and quality of the graphene-doped $\hbox {TiO}_2$ photoanodes. The Raman spectra provide detailed information on the vibrational modes of the materials, allowing us to identify characteristic peaks of anatase $\hbox {TiO}_2$ and graphene. The ID/IG ratio, calculated from the Raman spectra, indicates the level of defects and disorder in the graphene structure. This analysis is crucial for understanding the structural integrity and quality of the graphene used in the photoanodes, which directly impact their performance in DSSCs.

**Fig. 11:**
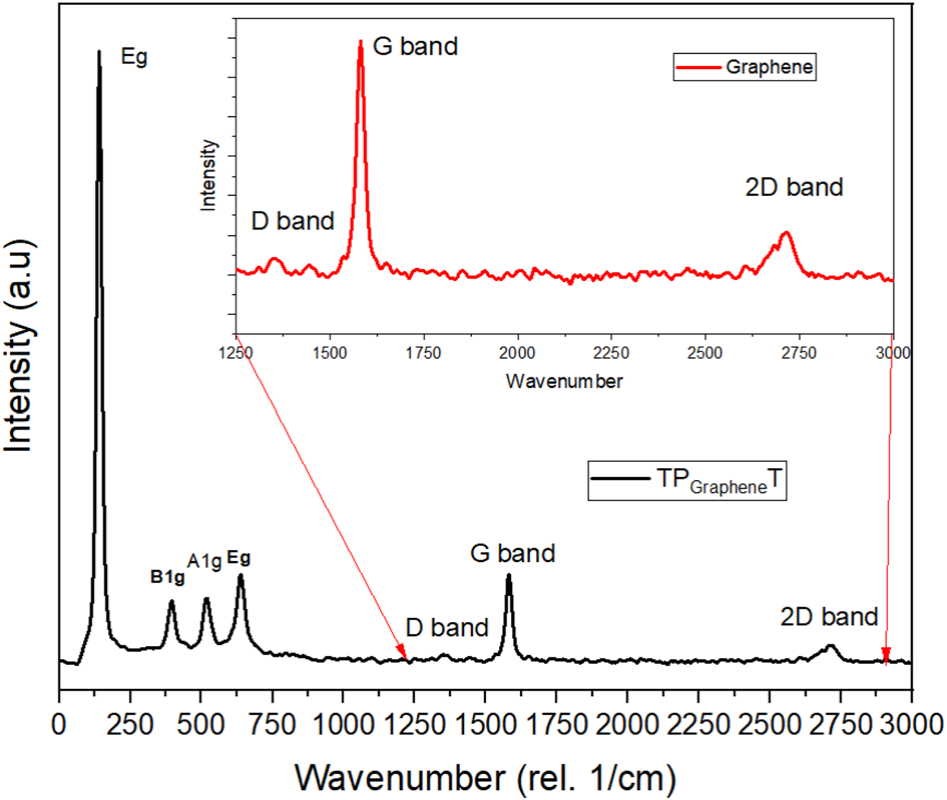
Raman spectrum of graphene-doped $\hbox {TiO}_2$ highlighting anatase $\hbox {TiO}_2$ and graphene peaks.

The Raman spectroscopic analysis of the graphene-doped $\hbox {TiO}_2$ photoanodes, integral to our study’s DSSCs, reveals critical insights into the composite material’s structural properties. As illustrated in Figure 11, distinct peaks corresponding to both the anatase phase of $\hbox {TiO}_2$ and graphene were identified, confirming the successful integration of graphene. Notably, the anatase $\hbox {TiO}_2$ peaks at approximately 144 $\hbox {cm}^{-1}$ (*Eg*), 399 $\hbox {cm}^{-1}$ (*B1g*), and 515 $\hbox {cm}^{-1}$ (*A1g/B1g*) indicate the presence of a photocatalytically active anatase phase. The graphene characterisation yielded specific peaks at around 1350 $\hbox {cm}^{-1}$ (*D band*), 1582 $\hbox {cm}^{-1}$ (*G band*), and 2670 $\hbox {cm}^{-1}$ (*2D band*). The ID/IG ratio, calculated as 0.94 from the intensities 529.8 (D) and 502.07 (G), suggests moderate disorder within the graphene structure. This degree of disorder indicates the presence of defects in graphene, which can enhance photocatalytic activity by providing additional sites for electron trapping, thereby facilitating charge separation and reducing recombination rates. Moreover, the G band’s presence confirms the integration of $\hbox {sp}^2$ carbon atoms, characteristic of graphene’s conducive electrical properties. The 2D band, associated with graphene’s layered structure, provides insights into the thickness and quality of graphene layers. This comprehensive analysis underscores the synergistic effect between graphene and $\hbox {TiO}_2$, attributing the observed enhancements in DSSC performance to improved electron mobility and light absorption capabilities.

Thus, the detailed Raman spectroscopy data (Figure 11) not only validate the successful doping of graphene into the $\hbox {TiO}_2$ matrix but also illuminate how graphene’s structural characteristics particularly the moderate disorder signified by the ID/IG ratio contribute to the enhanced efficiency of DSSC. This correlation between graphene’s structural properties and DSSC performance underpins the potential of material engineering in advancing photovoltaic technologies.

## Conclusion

This study highlights significant advancements in the efficiency and stability of bifacial dye-sensitized solar cells (DSSCs) by incorporating graphene into the SFF configuration of $\hbox {TiO}_{2}$ photoanodes. The optimal doping concentration of 0.1 wt% graphene achieved the highest total power conversion efficiency (PCE) of 11.04% under both front and back illumination on the fourth day, and 10.31% after stabilizing on the tenth day. This represents a considerable improvement compared to undoped $\hbox {TiO}_{2}$ photoanodes.

Graphene doping enhances stability over time by reducing electron-hole recombination and improving electron transport within the photoanodes. The sample doped with 0.1 wt% graphene ($\hbox {TP}_{0.1}$T) demonstrated high stability along with high efficiency after the initial performance evaluation. Additionally, the short-circuit current density ($\hbox {J}_{SC}$) for this concentration reached 17.04 mA/$\hbox {cm}^2$, compared to 15.99 mA/$\hbox {cm}^2$ for the standard TPT configuration. Moreover, Electrochemical impedance spectroscopy (EIS) confirmed that graphene decreases charge transfer resistance and extends the electron lifetime to 63.4 $\mu$s, contributing to the observed improvements. Characterization techniques such as field emission scanning electron microscopy (FESEM), energy-dispersive X-ray spectroscopy (EDS), X-ray diffraction (XRD), and Raman spectroscopy verified the successful integration of graphene into the $\hbox {TiO}_{2}$ matrix, maintaining its anatase structure while enhancing electron mobility and light-harvesting capabilities. Furthermore, Ultraviolet-visible (UV-vis) spectroscopy results mirrored the outcomes of the current-voltage (J-V) curve and EIS, showing a reduction in bandgap for the graphene-doped TPT configuration. These findings underscore the potential of graphene-doped $\hbox {TiO}_{2}$ photoanodes to advance bifacial DSSC technology, making it viable for practical applications, including building-integrated photovoltaics (BIPVs). Future research should focus on refining the doping process, exploring other doping materials, and conducting long-term performance evaluations in real-world conditions. Overall, doping $\hbox {TiO}_{2}$ photoanodes with graphene provides a promising strategy for enhancing the performance of bifacial DSSCs, contributing to the development of highly efficient and durable solar energy conversion devices.

## Supplementary Information

Supplementary Information.

## Data Availability

The datasets generated and/or analysed during the current study are available from the corresponding author on reasonable request.

## Materials availability

All materials used in this study are commercially available and have been described in the methodology section.

## References

[1] Baby, R., Nixon, P. D., Kumar, N. M., Subathra, M. S. P. & Ananthi, N. A comprehensive review of dye-sensitized solar cell optimal fabrication conditions, natural dye selection, and application-based future perspectives. *Environ. Sci. Pollut. Res.* **29**(1), 371–404. 10.1007/s11356-021-16976-8 (2022). 10.1007/s11356-021-16976-8 34674131

[2] Muñoz-García, A. B. et al. Dye-sensitized solar cells strike back. *Chem. Soc. Rev.* **50**(22), 12450–12550. 10.1039/D0CS01336F (2021). 34590638 10.1039/d0cs01336f PMC8591630

[3] Grätzel, M. Dye-sensitized solar cells. *J. Photochem. Photobiol. C Photochem. Rev.* **4**(2), 145–153. 10.1016/S1389-5567(03)00026-1 (2003).

[4] Kalyanasundaram, K. Applications of functionalized transition metal complexes in photonic and optoelectronic devices. *Coord. Chem. Rev.* **177**(1), 347–414. 10.1016/S0010-8545(98)00189-1 (1998).

[5] Chou, J.-C. et al. Graphene quantum dots as a co-sensitizer with improving light absorption for dye-sensitized solar cells. *IEEE Trans. Nanotechnol.* **22**, 20–27. 10.1109/TNANO.2023.3235335 (2023).

[6] Ji, J., Zhou, H., Eom, Y. K., Kim, C. H. & Kim, H. K. 1.42 % efficiency dye-sensitized solar cells by co-sensitizing novel thieno[3,2-b]indole-based organic dyes with a promising porphyrin sensitizer. *Adv. Energy Mater.* **10**(15), 2000124. 10.1002/aenm.202000124 (2020).

[7] Mary, C. I., Senthilkumar, M., Manobalaji, G. & Babu, S. M. Surface-treated cu2znsns4 nanoflakes as pt-free inexpensive and effective counter electrode in dssc. *J. Mater. Sci. Mater. Electron.* **31**(20), 18164–18174. 10.1007/s10854-020-04365-9 (2020).

[8] Mujahid, M. & Al-Hartomy, O. A. The effects of pt-doped tio2 nanoparticles and thickness of semiconducting layers at photoanode in the improved performance of dye-sensitized solar cells. *Materials* **15**(22), 7941. 10.3390/ma15227941 (2022). 36431427 10.3390/ma15227941 PMC9696509

[9] Chou, J.-C. et al. Photovoltaic properties of the titanium dioxide compact layer and reduced graphene oxide for dye-sensitized solar cells under different light intensities. *IEEE J. Photovolt.* **13**(1), 87–94. 10.1109/JPHOTOV.2022.3229189 (2023).

[10] Javed, H. M. A. et al. Investigations of anodization parameters and treatments on nanostructures for highly optimized dye-sensitized solar cells. *Surf. Interfaces* **27**, 101578. 10.1016/j.surfin.2021.101578 (2021).

[11] Qureshi, A. A. et al. Incorporation of zr-doped nanoparticles in electron transport layer for efficient planar perovskite solar cells. *Surf. Interfaces* **25**, 101299. 10.1016/j.surfin.2021.101299 (2021).

[12] Chou, H.-T. et al. Fabrication and properties of graphene electron multiple transporting layers for dye-sensitized solar cell. *IEEE J. Photovolt.* **11**(4), 850–857. 10.1109/JPHOTOV.2021.3075321 (2021).

[13] Van Le, C. et al. Rapidly forming the chemical bond titania-carbon in hybrid composite tio2/reduced graphene oxide to enhance the efficiency of dye-sensitized solar cells. *Arab. J. Sci. Eng.* **47**(1), 387–395. 10.1007/s13369-021-05462-5 (2022).

[14] Javed, H. M. A. et al. Encapsulation of nanotubes with cs nanoparticles to enhance electron injection and thermal stability of perovskite solar cells. *Surf. Interfaces* **23**, 101033. 10.1016/j.surfin.2021.101033 (2021).

[15] Park, J., Lee, P., Ko, M. J. Design and fabrication of long-term stable dye-sensitized solar cells: Effect of water contents in electrolytes on the performance. *Int. J. Precis. Eng. Manuf. Green Technol.* **6**(1), 125–131. 10.1007/s40684-019-00025-4 (2019).

[16] Ma’alinia, A., Moghaddam, H. A., Nouri, E. & Mohammadi, M. R. Long-term stability of dye-sensitized solar cells using a facile gel polymer electrolyte. *New J. Chem.* **42**(16), 13256–13262. 10.1039/C8NJ02157K (2018).

[17] Sonai, G. G., Tiihonen, A., Miettunen, K., Lund, P. D. & Nogueira, A. F. Long-term stability of dye-sensitized solar cells assembled with cobalt polymer gel electrolyte. *J. Phys. Chem. C* **121**(33), 17577–17585. 10.1021/acs.jpcc.7b03865 (2017).

[18] Storck, J. et al. Long-term stability improvement of non-toxic dye-sensitized solar cells via poly(ethylene oxide) gel electrolytes for future textile-based solar cells. *Polymers* **12**(12), 3035. 10.3390/polym12123035 (2020). 33353004 10.3390/polym12123035 PMC7766716

[19] Francis, M. K. et al. Bifacial dssc fabricated using low-temperature processed 3d flower like mos2 - high conducting carbon composite counter electrodes. *Mater. Today Commun.* **27**, 102208. 10.1016/j.mtcomm.2021.102208 (2021).

[20] Kang, J. S. et al. Highly efficient bifacial dye-sensitized solar cells employing polymeric counter electrodes. *ACS Appl. Mater. Interfaces* **10**(10), 8611–8620. 10.1021/acsami.7b17815 (2018). 29485266 10.1021/acsami.7b17815

[21] Rong, Y., Ku, Z., Li, X. & Han, H. Transparent bifacial dye-sensitized solar cells based on an electrochemically polymerized organic counter electrode and an iodine-free polymer gel electrolyte. *J. Mater. Sci.* **50**(10), 3803–3811. 10.1007/s10853-015-8945-9 (2015).

[22] Ngidi, N. P. D., Muchuweni, E. & Nyamori, V. O. Enhanced performance by heteroatom-doped reduced graphene oxide-tio2-based nanocomposites as photoanodes in dye-sensitised solar cells. *Int. J. Energy Res.* **46**(10), 13670–13686. 10.1002/er.8087 (2022).

[23] Rahman, M. Y. A. Review of graphene and its modification as cathode for dye-sensitized solar cells. *J. Mater. Sci. Mater. Electron.* **32**(19), 23690–23719. 10.1007/s10854-021-06898-z (2021).

[24] Cai, H. et al. Nanostructured composites of one-dimensional tio2 and reduced graphene oxide for efficient dye-sensitized solar cells. *J. Alloys Compd.* **697**, 132–137. 10.1016/j.jallcom.2016.10.189 (2017).

[25] Elseman, A. M.: Solar Cells: Theory, Materials and Recent Advances. BoD - Books on Demand (2021). 10.5772/intechopen.87735.

[26] AlSultan, H. A. et al. Advancing dye-sensitized solar cell performance with bifacial illumination: A novel stack formation framework approach. *Opt. Mater.* **153**, 115535. 10.1016/j.optmat.2024.115535 (2024).

[27] Srivastava, A., Tripathi, M., Awasthi, K. & Banerjee, S. *Materials Science: A Field of Diverse Industrial Applications* (Bentham Science Publishers, 2023).

[28] Sangwan, B. et al. Modified poly(vinyl alcohol) based polymer electrolyte for dye sensitized solar cells (dsscs). *Macromol. Symp.* **407**(1), 2100462. 10.1002/masy.202100462 (2023).

[29] Jia, H.-L., Zhang, M.-D., Yan, W., Ju, X.-H. & Zheng, H.-G. Effects of structural optimization on the performance of dye-sensitized solar cells: Spirobifluorene as a promising building block to enhance voc. *J. Mater. Chem. A* **4**(30), 11782–11788. 10.1039/C6TA03740B (2016).

[30] Neale, N. R. & Kopidakis, N. Tailoring the Interface to Improve Voc in Dye-Sensitized Solar Cells (2005)

[31] Abdulkarim, S. S., Hamad, A. E. & Hamad, F. K. Performance improvement of dye sensitized solar cell using vanadium (v) oxide as additives. *Libyan Int. J.* **69**, 4254. 10.37376/glj.vi69.4254 (2023).

[32] Guo, Z. et al. Voc over 1.4 v for amorphous tin-oxide-based dopant-free cspbi2br perovskite solar cells. *J. Am. Chem. Soc.* **142**(21), 9725–9734. 10.1021/jacs.0c02227 (2020). 32357007 10.1021/jacs.0c02227

[33] Ranjan, R., Aggarwal, S. & Bagga, A. Effect of interfacial resistances at tio2/tco/electrolyte interfaces on dye sensitized solar cells 1–4 (2012). 10.1109/ICEmElec.2012.6636274.

[34] Trang, T. T. et al. Effect of conducting ability of electrolytes on the photovoltaic performance of quasi-solid state dye-sensitized solar cells. *Mol. Cryst. Liq. Cryst.* **538**(1), 298–303. 10.1080/15421406.2011.564477 (2011).

[35] Razak, M. F. S. A. et al. Graphene-modified tio2 as photoanode using agarose gel electrolyte for dye-sensitized solar cell. *AIP Conf. Proc.* **2496**(1), 020004. 10.1063/5.0091009 (2022).

[36] Hora, C., Santos, F., Sales, M. G. F., Ivanou, D. K. & Mendes, A. Conventional and back-illuminated cobalt- and iodine-mediated dye-sensitized solar cells for artificial and solar light conversion. *ACS Appl. Energy Mater.* **5**(12), 14846–14857. 10.1021/acsaem.2c02307 (2022).

[37] 7.379 % power conversion efficiency of a numerically simulated solid-state dye-sensitized solar cell with copper (i) thiocyanate as a hole conductor. *East Eur. J. Phys.* 19–31 (2022) 10.26565/2312-4334-2022-3-03.

[38] Rajaramanan, T., Heidari Gourji, F., Velauthapillai, D., Ravirajan, P. & Senthilnanthanan, M. Enhanced photovoltaic properties of dye-sensitized solar cells through ammonium hydroxide-modified (nitrogen-doped) titania photoanodes. *Int. J. Energy Res.* **2023**(1), 1090174. 10.1155/2023/1090174 (2023).

[39] Fattah, A., Abbasi, A., Bavir, M. & Orouji, A. A. Anode resistance reduction of dye-sensitized solar cells using graphene for efficiency improvement. *Opt. Quantum Electron.* **53**(5), 247. 10.1007/s11082-021-02859-2 (2021).

[40] Apostolaki, M.-A. et al. Graphene quantum dot-tio2 photonic crystal films for photocatalytic applications. *Nanomaterials* **10**(12), 2566. 10.3390/nano10122566 (2020). 33371303 10.3390/nano10122566 PMC7766274

[41] Mehta, M., Chandrabose, G., Krishnamurthy, S., Avasthi, D. K. & Chowdhury, S. Improved photoelectrochemical properties of tio2-graphene nanocomposites: Effect of defect induced visible light absorption and graphene conducting channel for carrier transport. *Appl. Surf. Sci. Adv.* **11**, 100274. 10.1016/j.apsadv.2022.100274 (2022).

[42] Özönder, ., Ünlü, C., Güleryüz, C. & Trabzon, L. Doped graphene quantum dots uv-vis absorption spectrum: A high-throughput tddft study. *ACS Omega* **8**(2), 2112–2118. 10.1021/acsomega.2c06091 (2023). 10.1021/acsomega.2c06091 PMC9850463 36687068

[43] Phuthu, L., Dima, R. S., Maluta, N. E., Kirui, J. K. & Maphanga, R. R. Dft study of tio2 brookite (210) surface doped with silver and molybdenum. *Mater. Res. Express* **9**(9), 095901. 10.1088/2053-1591/ac8ae4 (2022).

