# Supplementary material for: Optimising bifacial dye-sensitized solar cells with graphene-enhanced TPT configuration photoanode for operational stability
Source: Sci Rep. 2025 Nov 3;15:38285. doi: 10.1038/s41598-025-06097-4 (PMC12583779; doi:10.1038/s41598-025-06097-4)
Supplement: Supplementary file 1 — Supplementary Information. [file 41598_2025_6097_MOESM1_ESM.pdf]

Supplementary Information Document

Hussein A. AlSultan[1,2*], Suhaidi Shafie[1,2*], Mohd Nizar Hamidon[1,2], Ismayadi Ismail[2], Shyam S. Pandey[3], Fauzan Ahmad[4]

[1*]Institute of Nanoscience and Nanotechnology (ION2), Universiti Putra Malaysia, Jalan UPM, Serdang, 43400, Selangor, Malaysia.
[2]Faculty of Engineering, Universiti Putra Malaysia, Jalan UPM, Serdang, 43400, Selangor, Malaysia.
[3]Department of Biological Functions Engineering, Kyushu Institute of Technology, Sensui-cho, Kitakyushu, 804-8550, Fukuoka, Japan.
[4]Malaysia-Japan International Institute of Technology, Universiti Teknologi Malaysia, , Kuala Lumpur, 54100, Wilayah Persekutuan, Malaysia.

*Corresponding author(s). E-mail(s): gs58052@student.upm.edu.my;hussein.a.alsultan@gmail.com; suhaidi@upm.edu.my;
Contributing authors: mnh@upm.edu.my; ismayadi@upm.edu.my; shyam@life.kyutech.ac.jp; fauzan.kl@utm.my;

1 Supplementary Information

1.1 TiO_2 Paste Preparation and Graphene Doping

The fabrication of the photoanode paste commenced with the careful preparation of an ethanol-ethyl cellulose mixture. This was achieved by dissolving 5 grams of ethyl cellulose into 10 ml of ethanol, stirring at 500 rpm in a controlled environment to avoid lumps, ensuring a smooth, homogeneous mixture over two hours. Simultaneously, the TiO_2 powder preparation was meticulously conducted by heating 1 gram of TiO_2 P25 at 100°C to eradicate residual moisture, optimizing the powder's reactivity. Following this, the acid addition involved a calculated introduction of 0.16 ml of acetic acid to the warm TiO_2, initiating a controlled pre-aggregation phase. The water integration step saw the careful addition of 0.8 ml deionized water, then methodically augmenting

the mixture with 10 ml of ethanol in 2 ml increments, ensuring thorough dispersion of constituents.

The process advanced with final additions, introducing another 10 ml of ethanol already mixed with the desired percentage of graphene and ultrasonicated from the start to ensure continuous dispersion of graphene in the solvent, and 3.5 ml of α-terpineol, intensifying the stirring for enhanced blend uniformity. The mixture underwent ultrasonication, employing high-frequency ultrasonic waves for one hour to break down particle agglomerates. The concluding phase, extended stirring and concentration, integrated the ultrasonicated solution with the ethanol-ethyl cellulose mixture for 12 hours of stirring, followed by a meticulous evaporation process until the volume reduced to a tenth, achieving the requisite paste viscosity for photoanode application.

1.2 Electrolyte and Counter Electrode Preparation

The electrolyte solution was synthesized using specific concentrations of the following chemicals:

- Acetonitrile (Solvent): 5 ml
- Iodine (I_2): 0.05 M (64 mg)
- Lithium Iodide (LiI): 0.1 M (67 mg)
- 4-tert-Butylpyridine (tBP): 0.5 M (0.4 ml)
- Ethyl-methyl-imidazolium Iodide (EMII): 0.6 M (798 mg)

These components were mixed in a 10 ml beaker and stirred with a magnetic stirrer for 15 minutes to ensure thorough mixing and complete dissolution of the solid components. The mixture was then transferred into a 10 ml well-sealed bottle to prevent evaporation and degradation of the electrolyte solution. This prepared electrolyte was subsequently injected into the dye-sensitized solar cells through pre-drilled holes in the counter electrode substrates, ensuring proper filling and contact with the photoanode and counter electrode.

1.3 Counter Electrodes Preparation

The counter electrodes were prepared using a ready-to-use platinum (Pt) solution from Solaronix. The preparation involved the following steps:

1. **Cleaning of FTO Substrates:** The FTO glass substrates were cleaned thoroughly using an ultrasonic bath with acetone, isopropyl alcohol (IPA), and deionized water, each for 15 minutes. The cleaned substrates were then dried with a nitrogen stream and treated with UV-ozone for 15 minutes to remove any organic contaminants.
2. **Application of Pt Solution:** The ready-to-use Pt solution was applied to the cleaned FTO substrates using a spin-coating technique.
3. **Spin Coating and Annealing:** 50 L of the Pt solution was deposited onto the FTO substrate, which was then spin-coated at 1500 rpm for 10 seconds to form a thin, uniform layer. The spin-coated substrates were then annealed at 450C for 15

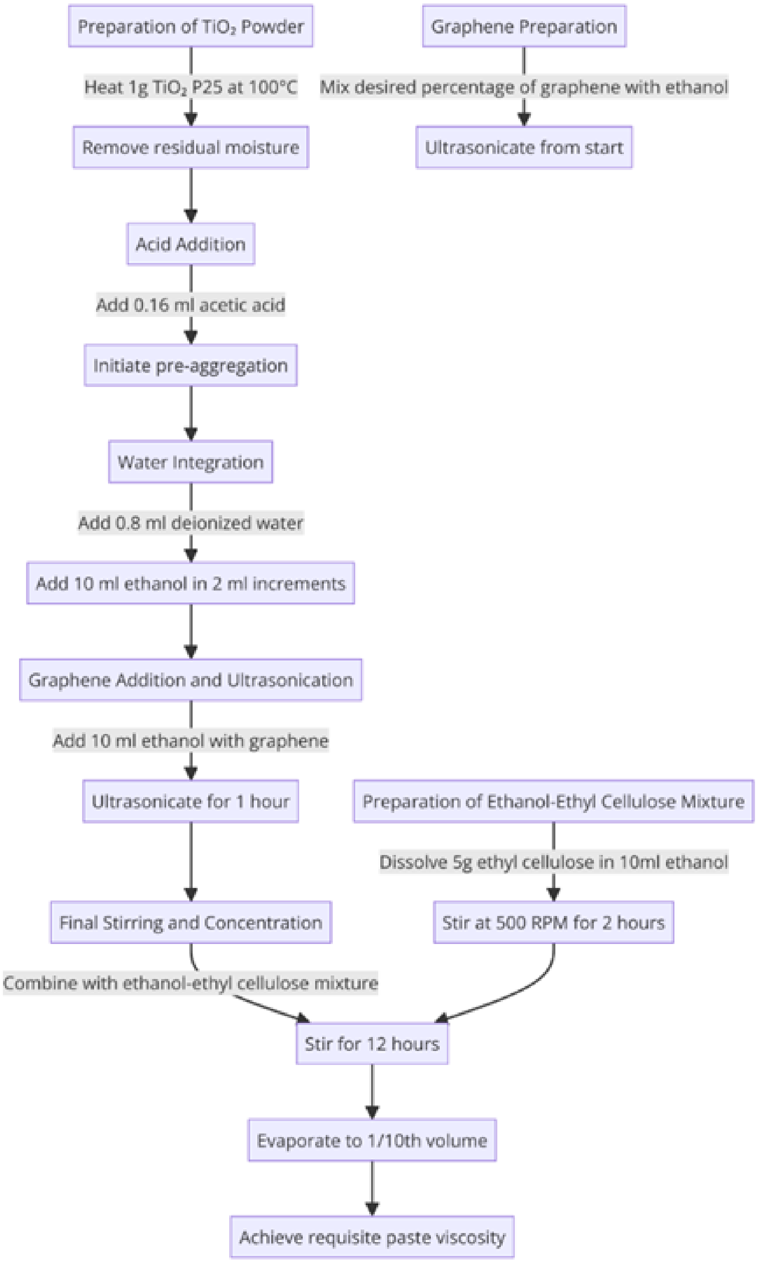

Fig. 1: Process flow diagram for TiO_2 paste preparation and graphene doping.

minutes to form a platinum layer, enhancing the catalytic activity and conductivity of the counter electrode.

2 Equations and Calculations

2.1 Power Conversion Efficiency (PCE)

The Power Conversion Efficiency (PCE) of dye-sensitized solar cells (DSSCs) is a crucial metric that quantifies the effectiveness of the solar cells in converting sunlight into electrical energy. It is calculated using the formula:

$$\text{PCE (\%)} = \left(\frac{\text{Maximum Power Output } (P_{\text{max}})}{\text{Incident Light Power } (P_{\text{in}})} \right) \times 100. \tag{1}$$

Where P_{max} is the maximum power output from the solar cell, obtained from the product of the current density (J) and the voltage (V) at the maximum power point on the current-voltage (J-V) curve. P_{in} is the power of the incident light per unit area, calculated by multiplying the intensity of the incident light (in watts per square meter) by the active area of the solar cell (in square meters). In our experiments, the J-V characteristics of the cells were measured under standard test conditions (STC), which include a light intensity of 1000 W/m^2 , simulating sunlight at AM 1.5G. The PCE values were then tracked over time to assess the stability and efficiency of different graphene concentrations in the TiO_2 photoanodes, under both front and back illumination conditions. This quantitative measure helps in evaluating the enhancements achieved through the strategic incorporation of graphene, aimed at optimizing the photovoltaic performance of bifacial DSSCs.

2.2 Analysis of PCE Trends Over Time Using Exponential Equations

To comprehensively understand the stability and performance trends of our dye-sensitized solar cells (DSSCs) over an extended period, we employed exponential equations to model the decay in Power Conversion Efficiency (PCE) over time. This methodological approach allows us to predict long-term trends from short-term experimental data, providing insights into the durability and degradation patterns of the solar cells under test conditions. The exponential model used is given by:

$$\text{PCE}(t) = \text{PCE}_0 \times e^{-kt}. \tag{2}$$

where $\text{PCE}(t)$ is the PCE at time t, PCE_0 is the initial PCE measured, and k is the decay constant, which represents the rate of efficiency loss over time. The values for k and the coefficient of determination, R^2, are determined through curve fitting techniques on the experimental data obtained from initial and periodic J-V measurements under standard test conditions. The R^2 value helps in assessing the fit of the exponential model to the data, providing a quantitative measure of how well the model captures the observed trends. This analysis not only highlights the robustness of the graphene-enhanced TiO_2 photoanodes in maintaining efficiency over time but also aids in identifying the optimal graphene concentration that offers the best balance between initial performance and long-term stability. By quantifying the degradation rate and

verifying the fit of our model via R^2, we can infer the impact of various concentrations of graphene and other experimental conditions on the lifespan and effectiveness of bifacial DSSCs.

2.3 Electrochemical Impedance Spectroscopy (EIS) Analysis and Calculations

For our dye-sensitized solar cells (DSSCs), Electrochemical Impedance Spectroscopy (EIS) was utilized to evaluate the electrochemical properties and interface characteristics of the graphene-enhanced TiO_2 photoanodes. The EIS measurements were conducted using a frequency range suitable for solar cell analysis, and the results were visualized through Nyquist and Bode plots generated using ZView software. The Nyquist plot, characterized by its semicircle, was analyzed by fitting it to an equivalent circuit model to derive key parameters: the series resistance (R_S) and the charge transfer resistance (R_{CT}).

The equivalent circuit typically includes elements representing the resistive and capacitive properties of the cell, where R_S corresponds to the inherent resistance of the materials and contacts, and R_{CT} represents the resistance to charge transfer at the electrode/electrolyte interface. The fitting process involves adjusting the circuit parameters until the simulated impedance curve aligns with the experimental data, ensuring an optimal fit that is quantified by the chi-squared value (χ^2).

Furthermore, the electron lifetime (τ_e) was calculated from the Bode plot, which provides the phase angle versus frequency data. The minimum phase angle frequency ($f_{\min}$) was identified, corresponding to the peak of the imaginary part of impedance (Z''), and τ_e was calculated using the relationship:

$$\tau_e = \frac{1}{2\pi f_{\min}}. \tag{3}$$

This calculation reflects the time constant for electron recombination, providing insights into the efficiency of charge separation and the stability of the charge carriers within the DSSC. The combination of R_S, R_{CT}, and τ_e offers a comprehensive view of the cell's electrochemical performance, highlighting the impact of graphene incorporation on improving the electron transport and reducing recombination rates, which are critical for enhancing the overall efficiency and stability of DSSCs.

2.4 Tauc Plot Calculations from UV-Vis Data

The Tauc plot is a critical tool used to estimate the bandgap energy of semiconductor materials, such as the graphene-enhanced TiO_2 used in our dye-sensitized solar cells (DSSCs). For this analysis, the optical absorption data obtained from UV-Visible spectroscopy was used to construct Tauc plots, which relate the photon energy to the absorption edge of the material.

The optical absorption coefficient (α) is determined from the UV-Vis absorbance data using the formula:

$$\alpha = \frac{2.303 \times A}{d}. \quad (4)$$

where A is the absorbance and d is the thickness of the sample. Once α is calculated, the Tauc equation for direct bandgap semiconductors can be applied:

$$(\alpha h\nu)^2 = A(h\nu - E_g). \quad (5)$$

In this equation, $h\nu$ represents the photon energy, E_g is the optical bandgap energy, and A is a constant. The plot of $(\alpha h\nu)^2$ versus $h\nu$ allows us to determine the bandgap energy by extrapolating the linear portion of the plot to intersect the energy axis $h\nu$ at $(\alpha h\nu)^2 = 0$.

3 Performance Parameters Tables

Table 1: Performance parameters of DSSCs with the TPT configuration under front illumination from day 0 to day 10.

Days	J_{SC} [mA/cm^2]	V_{OC} [V]	FF	PCE [%]	J_{max} [mA/cm^2]	V_{max} [V]
0	15.99	0.68	0.57	6.16	13.99	0.44
1	13.98	0.73	0.57	5.85	12.19	0.48
2	14.84	0.73	0.61	6.58	13.16	0.50
3	14.75	0.74	0.61	6.58	13.16	0.50
4	14.91	0.74	0.61	6.71	13.42	0.50
5	14.60	0.75	0.61	6.66	13.14	0.51
6	14.29	0.76	0.61	6.60	12.88	0.51
7	13.99	0.76	0.61	6.55	12.61	0.52
8	13.70	0.77	0.61	6.50	12.35	0.53
9	13.41	0.78	0.62	6.45	12.10	0.53
10	13.13	0.79	0.62	6.40	11.85	0.54

Table 2: Performance parameters of DSSCs with the TPT configuration under back illumination from day 0 to day 10.

Days	J_{SC} [mA/cm^2]	V_{OC} [V]	FF	PCE [%]	J_{max} [mA/cm^2]	V_{max} [V]
0	9.32	0.65	0.63	3.81	8.48	0.45
1	8.40	0.71	0.67	4.00	7.68	0.52
2	8.48	0.71	0.67	4.05	7.78	0.52
3	9.56	0.72	0.66	4.49	8.72	0.51
4	10.49	0.72	0.64	4.86	9.53	0.51
5	9.72	0.73	0.65	4.62	8.86	0.52
6	9.01	0.74	0.66	4.38	8.23	0.53
7	8.35	0.74	0.67	4.16	7.65	0.54
8	7.73	0.75	0.68	3.95	7.11	0.56
9	7.17	0.76	0.69	3.75	6.61	0.57
10	6.64	0.77	0.70	3.56	6.14	0.58

Table 3: Performance parameters of DSSCs with the $TP_{0.05}T$ configuration under front illumination from day 0 to day 10.

Days	J_{SC} [mA/cm^2]	V_{OC} [V]	FF	PCE [%]	J_{max} [mA/cm^2]	V_{max} [V]
0	14.96	0.68	0.55	5.61	13.04	0.43
1	15.37	0.67	0.56	5.77	13.42	0.43
2	16.80	0.72	0.54	6.50	14.44	0.45
3	15.12	0.72	0.59	6.42	13.27	0.48
4	14.28	0.73	0.63	6.59	12.68	0.52
5	13.99	0.74	0.63	6.50	12.41	0.52
6	13.70	0.75	0.62	6.40	12.15	0.53
7	13.41	0.76	0.62	6.30	11.90	0.53
8	13.14	0.77	0.61	6.21	11.65	0.53
9	12.87	0.78	0.61	6.12	11.40	0.54
10	12.60	0.79	0.61	6.03	11.16	0.54

Table 4: Performance parameters of DSSCs with the $TP_{0.05}T$ configuration under back illumination from day 0 to day 10.

Days	J_{SC} [mA/cm^2]	V_{OC} [V]	FF	PCE [%]	J_{max} [mA/cm^2]	V_{max} [V]
0	8.07	0.66	0.64	3.40	7.23	0.47
1	8.80	0.71	0.66	4.12	7.92	0.52
2	8.71	0.71	0.68	4.20	7.92	0.53
3	8.46	0.72	0.67	4.08	7.62	0.53
4	8.26	0.73	0.66	4.00	7.40	0.54
5	8.06	0.73	0.67	3.96	7.25	0.55
6	7.87	0.74	0.68	3.93	7.11	0.55
7	7.69	0.74	0.68	3.90	6.97	0.56
8	7.51	0.75	0.69	3.87	6.83	0.57
9	7.33	0.75	0.69	3.84	6.70	0.57
10	7.16	0.76	0.70	3.81	6.56	0.58

Table 5: Performance parameters of DSSCs with the $TP_{0.1}T$ configuration under front illumination from day 0 to day 10.

Days	J_{SC} [mA/cm^2]	V_{OC} [V]	FF	PCE [%]	J_{max} [mA/cm^2]	V_{max} [V]
0	16.52	0.67	0.53	5.83	13.56	0.43
1	16.80	0.69	0.51	5.90	14.04	0.42
2	17.04	0.71	0.56	6.73	14.64	0.46
3	15.98	0.71	0.58	6.66	14.02	0.47
4	15.28	0.72	0.61	6.72	13.72	0.49
5	14.85	0.73	0.62	6.72	13.36	0.50
6	14.43	0.74	0.63	6.71	13.02	0.52
7	14.03	0.75	0.64	6.70	12.68	0.53
8	13.63	0.76	0.65	6.69	12.35	0.54
9	13.25	0.77	0.66	6.69	12.03	0.56
10	12.88	0.78	0.66	6.68	11.72	0.57

Table 6: Performance parameters of DSSCs with the $TP_{0.1}T$ configuration under back illumination from day 0 to day 10.

Days	J_{SC} [mA/cm^2]	V_{OC} [V]	FF	PCE [%]	J_{max} [mA/cm^2]	V_{max} [V]
0	9.15	0.64	0.58	3.38	8.05	0.42
1	8.49	0.70	0.66	3.91	7.67	0.51
2	9.68	0.70	0.62	4.17	8.70	0.48
3	9.59	0.71	0.62	4.20	8.66	0.48
4	9.84	0.71	0.62	4.36	8.90	0.49
5	9.36	0.72	0.63	4.23	8.47	0.50
6	8.92	0.73	0.63	4.10	8.05	0.51
7	8.49	0.73	0.64	3.98	7.66	0.52
8	8.08	0.74	0.64	3.86	7.29	0.53
9	7.70	0.75	0.65	3.74	6.93	0.54
10	7.33	0.76	0.65	3.63	6.60	0.55

Table 7: Performance parameters of DSSCs with the $TP_{0.2}T$ configuration under front illumination from day 0 to day 10.

Days	J_{SC} [mA/cm^2]	V_{OC} [V]	FF	PCE [%]	J_{max} [mA/cm^2]	V_{max} [V]
0	14.64	0.69	0.56	5.66	12.87	0.44
1	14.07	0.71	0.57	5.69	12.10	0.47
2	14.00	0.73	0.59	6.08	12.16	0.50
3	14.15	0.72	0.61	6.20	12.54	0.49
4	14.24	0.72	0.61	6.29	12.84	0.49
5	14.00	0.73	0.62	6.29	12.60	0.50
6	13.76	0.74	0.62	6.29	12.36	0.51
7	13.52	0.75	0.62	6.29	12.12	0.52
8	13.29	0.76	0.62	6.29	11.89	0.53
9	13.06	0.77	0.63	6.29	11.66	0.54
10	12.84	0.78	0.63	6.29	11.44	0.55

Table 8: Performance parameters of DSSCs with the $TP_{0.2}T$ configuration under back illumination from day 0 to day 10.

Days	J_{SC} [mA/cm^2]	V_{OC} [V]	FF	PCE [%]	J_{max} [mA/cm^2]	V_{max} [V]
0	7.44	0.63	0.51	2.40	6.32	0.38
1	7.54	0.64	0.51	2.46	6.15	0.40
2	7.09	0.65	0.58	2.66	6.19	0.43
3	6.94	0.71	0.62	3.05	6.17	0.50
4	6.72	0.76	0.69	3.51	6.16	0.57
5	6.64	0.76	0.69	3.51	6.11	0.57
6	6.56	0.76	0.70	3.51	6.06	0.58
7	6.48	0.76	0.71	3.52	6.01	0.58
8	6.40	0.77	0.72	3.52	5.96	0.59
9	6.33	0.77	0.72	3.52	5.91	0.59
10	6.25	0.77	0.73	3.52	5.87	0.60
